# Drought effects on the stability of forest-grassland ecotones under gradual climate change

**DOI:** 10.1371/journal.pone.0206138

**Published:** 2018-10-24

**Authors:** Ceres Barros, Wilfried Thuiller, Tamara Münkemüller

**Affiliations:** 1 Université Grenoble Alpes, Université Savoie Mont Blanc, CNRS, Laboratoire d’Écologie Alpine (LECA), Grenoble, France; 2 Department of Forest Resources Management, Faculty of Forestry, UBC Forest Sciences Centre, Vancouver BC, Canada; 3 Pacific Forestry Centre, Canadian Forest Service—Natural Resources Canada, Victoria BC, Canada; Chinese Academy of Forestry, CHINA

## Abstract

Plant communities in forest-grassland ecotones of the European Alps are already suffering from gradual climate change and will likely be exposed to more frequent and intense drought periods in the future. Yet, how gradual climate change and extreme drought will affect the stability of these plant communities is largely unknown. Here, we investigated how drought modulates the effects of gradual climate change on the long-term structural stability of these ecotone communities using a multidimensional approach. Using a spatially explicit landscape vegetation model, we simulated three drought scenarios, on top of gradual changes of climate variables, and their impacts on the dynamics of 24 plant functional groups, distinguishing between forests and grasslands, as well as different land uses. We then used *n*-dimensional hypervolumes to define community states under the different drought scenarios, and compared them to initial conditions to assess changes in community structural stability. In general, added drought effects did not counteract the long-term consequences of gradual climate changes, although they resulted in quantitatively different effects. Importantly, drought and climate change had non-negligible consequences for taxonomic and functional structure that differed between communities and land-use regimes. For instance, forest taxonomic structure was more overall more stable than grassland’s, despite the observed functional shifts towards more warm-adapted species compositions. Conversely, unmanaged grasslands were the least stable, suffering the loss of characteristic alpine species. Also, while frequent and severe drought regimes caused forests to become more variable in time, they had the opposite effect on grasslands. Our results agree with observations of drought- and climate-driven changes in mountain communities of the Alps, and we discuss their relevance for ecosystem management. Importantly, we demonstrate the utility of this multidimensional approach to study community stability for analysing cross-community and cross-disturbance responses to global change.

## Introduction

Climate change is expected to increase average temperatures, but also the frequency and intensity of drought events [1]. Since drought can have negative effects on plant growth and survival [2], changes in drought regimes have implications for plant community structure and composition [3], ultimately affecting ecosystem functioning and services [4]. Extreme droughts have already caused significant forest diebacks around the globe [5] and declines in plant productivity across Europe [6]. In the future, we can expect that even areas that have been “safe” from drought so far, like the European Alps, will suffer more frequent and intense drought events [1].

In the European Alps, forest-grassland ecotones at the treeline are sources of important ecosystems services [7,8]. Current treelines have resulted from centuries of agro-pastoral activities, whose abandonment causes forests to move upwards and encroach open grassland habitats [9,10]. The added effect of climate change further promotes this encroachment and causes the loss of suitable habitat for alpine grasslands at their lower range edges [10–12], as well as changes in taxonomic and functional diversity at the treeline itself [7,13]. How changes in drought regimes will modulate these impacts, however, is still poorly known. Depending on its frequency, intensity and temporal extent, drought may impact forest-grassland ecotones differently [3,14] and ultimately affect their stability. Simulation models suggest that drought can facilitate species adapted to warmer and drier climates, increasing woody encroachment at higher elevations over the short term, but cause forest expansion rates to slow down over the long term, when compared to climate and land-use changes alone [15].

However, communities are more than the simple sum of their species, and to better understand their stability we need to go beyond studying the responses of isolated community properties, like total biomass [16] or population densities of a species or group of species [17]. Instead, the responses of multiple facets of biodiversity (e.g. changes in taxonomic and functional structures and compositions) should provide a better image of the consequences of global change drivers for community stability [18–20]. Moreover, as different ecosystems respond differently to global change [21], adequate ecosystem management requires knowledge on their relative stability to interacting drivers.

Here, we sought to understand how drought modulates the effects of gradual climate change on the multidimensional structural stability of different plant communities and land-use practices. We focused our analysis on forest-grassland ecotone communities in the Alps, namely unmanaged forests, and managed and unmanaged grasslands. We use a landscape dynamic vegetation model (see Barros *et al*. [15]) to reproduce the effects of three different drought regimes (in combination with gradual changes in climate in bioclimatic variables) on the stability of these plant communities in the Écrins National Park (French Alps). This model simulates the spatio-temporal dynamics of plant functional groups (PFGs) and their responses to both environmental and land-use changes. We then analysed and compared the resulting states of communities of PFGs with *n*-dimensional hypervolumes in order to account for the multi-facetted nature of biodiversity, instead of focusing on the responses of single community variables [18]. This approach allows investigation of how environmental change affects both taxonomic and functional diversity and assessment of the relative stability of different communities. As in other stability studies, we considered communities to be stable if, when disturbed, they showed small departures from their initial states, and when temporal variance was lower [22,23]. This meant that communities were deemed more stable when their initial and final hypervolumes were most similar. This framework captures structural community changes in their whole, while still allowing to analyse how they translate into taxonomic and functional changes. Hence, rather than providing quantitative predictions of drought effects on particular community properties, we analysed how extreme drought modulates the impact of climate change on the multidimensional stability of plant communities in Alpine forest-grassland ecotones, explicitly considering effects of the ecosystem type and land-use regime.

## Materials and methods

### Study area

Located in Southeast France, in the French Alps, the Écrins National Park (NP) is characterised by strong elevational gradients (from 669 to 4102 m a.s.l.), which together with a diverse flora (ca. 2000 vascular plant species) generate a variety of plant communities, from lowland forests to nival communities, passing through wetlands, as well as schlerophylous vegetation. Around 68% of the park’s surface is currently managed, mainly for agriculture (grazing, 48%; crop fields and mown grasslands, 9.8%) and forestry (14%) [24].

### Vegetation dynamics model—FATE-HD

We simulated the effects of gradual climate change and drought regimes on the vegetation of the Écrins NP using the FATE-HD simulation platform. The implementation and parameterisation of FATE-HD is explained in detail in Boulangeat *et al*. [25] (base model), Boulangeat *et al*. [26] (gradual climate change) and Barros *et al*. [15] (added drought effects). Therefore, we only give an overview here. Vegetation dynamics in FATE-HD are the result of the explicit simulation of the population dynamics, dispersal, biotic interactions (through interaction for light), and response to management (mowing and grazing) and abiotic conditions of 24 plant functional groups (PFGs) on an annual basis. These PFGs represent the ca. 400 dominant species present in the park, grouped by functional similarity and tolerance to abiotic and biotic conditions. The park’s surface (ca. 270 000 ha) was divided into 100x100m grid pixels. In a pixel, PFG population dynamics are dependent on PFG demographic parameters, their ability to tolerate shade and the pixel’s habitat suitability. The amount of shade depends on the size and abundance of PFGs, which can occupy up to 5 vertical strata (see Table A in S3 Appendix) depending on their maximum height. The taller and more abundant a PFG becomes, the more shade it casts on smaller PFGs. Habitat suitability affects seed production and recruitment, and was calculated for each PFG using a species distribution modelling approach (*R* package *biomod2*; Thuiller *et al*. [27]), with slope, percentage of calcareous soil and five bioclimatic variables as predictors (isothermality, temperature seasonality, temperature annual range, mean temperature of coldest quarter and annual precipitation). Bioclimatic variables were averaged across 1961–1990 to calculate ‘current’ habitat suitability (see S1 Appendix). Short- and long-distance seed dispersal connect pixels and their vegetation dynamics. Effective dispersal distances are conditional on the dispersal capacity of each PFG.

### Simulating land use, gradual climate change and drought events

FATE-HD includes spatially-explicit modules for land use, gradual climate change and drought effects, which affect vegetation dynamic in terms of PFG mortality and regeneration (triggering resprouting and/or reducing seed production).

Land use is simulated in the form of mowing and grazing (the two most important agro-pastoral land-use activities in the park), which occur once a year in the areas that have been mapped in 2006 by the park managers. Mowing and grazing affect PFG survival or cause them to resprout, depending on a PFG’s age class, size and palatability in the case of grazing. Mowing also removes all trees taller than 1.5m (second height stratum). Hence, binary maps of mown areas and areas grazed at low, medium and high intensities were fed into the model to simulate their presence/absence per pixel. We kept grazing and mowing activities constant throughout all simulation scenarios to simulate the land management of the park as of 2006.

Gradual climate change is simulated as changes in habitat suitability, thus affecting seed production and recruitment. For each PFG, future habitat suitability maps were calculated based on the species distribution models described above, using future projections of the five bioclimatic variables as predictors (downscaled at 100 m resolution grid). For a direct comparison with results shown in Barros *et al*. [15], we used forecasts from the Intergovernmental Panel on Climate Change (IPCC) 4^th^ Assessment Report under the A1B emissions scenario for years 2020, 2050 and 2080 [28]. Resulting habitat suitability maps were interpolated between current (1961–1990 period) and 2020 projections, between 2020 and 2050 projections, and between 2050–2080 projections, to obtain smoother changes in climate.

Drought events were simulated using maps of pixel-based drought intensity (*Din*) values, calculated as the lowest monthly values of moisture index (*MI*) in each year. Because *MI* is measured as the difference in precipitation and evapotranspiration, negative *MI* values indicate climatic drought conditions. Therefore, the lower the *Din* value, the more severe the drought (see S1 Appendix for formulae). We calculated *Din* maps for ‘current’ and ‘future’ conditions. Current *Din* maps were based on *Din* values averaged across years 1961–1990 per pixel and were used to simulate “no drought years”. Future *Din* maps were based on *Din* predictions for 2080 (using climate projections following the A1B scenario), which we increased or decreased uniformly across the landscape to vary drought intensity. Future ‘moderate’ *Din* maps corresponded to a 20% increase of *Din* values relative to projections for 2080 (lower intensity), while future ‘severe’ *Din* maps corresponded to 20% decrease of the projected values (higher intensity; Fig B in S4 Appendix). Drought frequency was simulated by feeding future *Din* maps more or less frequently to FATE-HD. We considered two levels of frequency ‘sporadic’ and ‘frequent’, which were combined with different intensities to simulate different drought scenarios (see below).

The consequences of drought for PFGs depend both on the pixel *Din* value, but also on the PFG’s tolerance to drought conditions. Because the 24 PFGs represent a wide variety of plant species and several life forms, for many of which drought response traits are largely unknown, a trait-based parametrisation of PFG drought tolerance was impossible. Hence, parameters were statistically-derived, by comparing projected pixel *Din* values against PFG-specific historical *Din* values between 1961–1990 (i.e. distribution of *Din* values from locations where the PFG was present during this period, *Din*_*1961-1990*_ distribution). We assume that the lower a simulated pixel *Din* value is with respect to a PFG’s *Din*_*1961-1990*_ distribution, the more negatively drought affects that PFG. For each PFG two drought thresholds were calculated from its *Din*_*1961-1990*_ distribution to determine whether drought effects are moderate (pixel *Din <* $\bar{x}$ - 1.5SD of PFG’s *Din*_*1961-1990*_) or severe (pixel *Din <* $\bar{x}$ - 2.0SD of PFG’s *Din*_*1961-1990*_)–this means that the same pixel *Din* value can affect some PFGs more than others. Hence, at each time-step and in each pixel, FATE-HD compares the simulated pixel *Din* value against the drought thresholds of the PFGs present in that pixel. Moderate drought effects decrease PFG recruitment and fertility, while severe effects also reduce survival. It has been shown that even if drought is not extreme, repeated or prolonged drought conditions can ultimately reduce survival for drought sensitive species [29–31]. Also, extreme drought can continue to affect growth and survival throughout subsequent non-drought years [29,30,32]. Thus, FATE-HD includes two memory effects that aim to simulate the consequences of repeated drought events and post-drought effects: PFGs suffer severe drought effects when subjected to successive drought events, and tree and shrub PFGs suffer post-drought effects (higher mortality, lower recruitment and fertility) if they suffered severe drought effects during the previous year (herbaceous PFGs were assumed to fully recover in non-drought years [33,34]). PFG drought responses are further refined by conditioning them to the PFG’s life form, soil moisture requirements and age. Herbaceous PFGs are the most sensitive group, but also recover faster [33,34], while shrubs (C4 group) and phanerophyte PFGs are less sensitive to drought, but recover slower once affected by drought [35,36]. Younger and older PFGs, the extremes of the size gradient, are known to be more negatively affected by drought [29,37] and thus have higher drought-related mortality rates. Similarly, PFGs with higher soil moisture requirements should be less adapted to drought [35], and suffer higher reductions in recruitment, fertility and survival when affected by drought. Finally, the presence of an established canopy is known to exert a protective effect against drought conditions [38,39]. This is reproduced in FATE-HD by increasing a pixel’s *Din* value by 25% when tree cover is >40%. All these refinements of drought responses were developed and described in Barros *et al*. [15], based on literature information and expert knowledge.

Full lists of parameters referring to demography, dispersal, shade tolerance and grazing/mowing effects can be found in Boulangeat *et al*. [25], together with a detailed description for the calculation of habitat suitability maps (also in S1 Appendix). Details on PFG building have been described in Boulangeat *et al*. [40]. Drought parameter lists can be found in Barros *et al*. [15], together with their validation (also in S1 Appendix). For PFGs’ species and trait values, see Tables A and B in S3 Appendix, and for a brief description of PFGs see Table A in S3 Appendix. Climate data sources and formulae for the calculation of *Din*, and details regarding PFG-specific *Din* distributions and soil moisture requirements are also described in S1 Appendix.

### Simulation experiment

**Fig 1:**
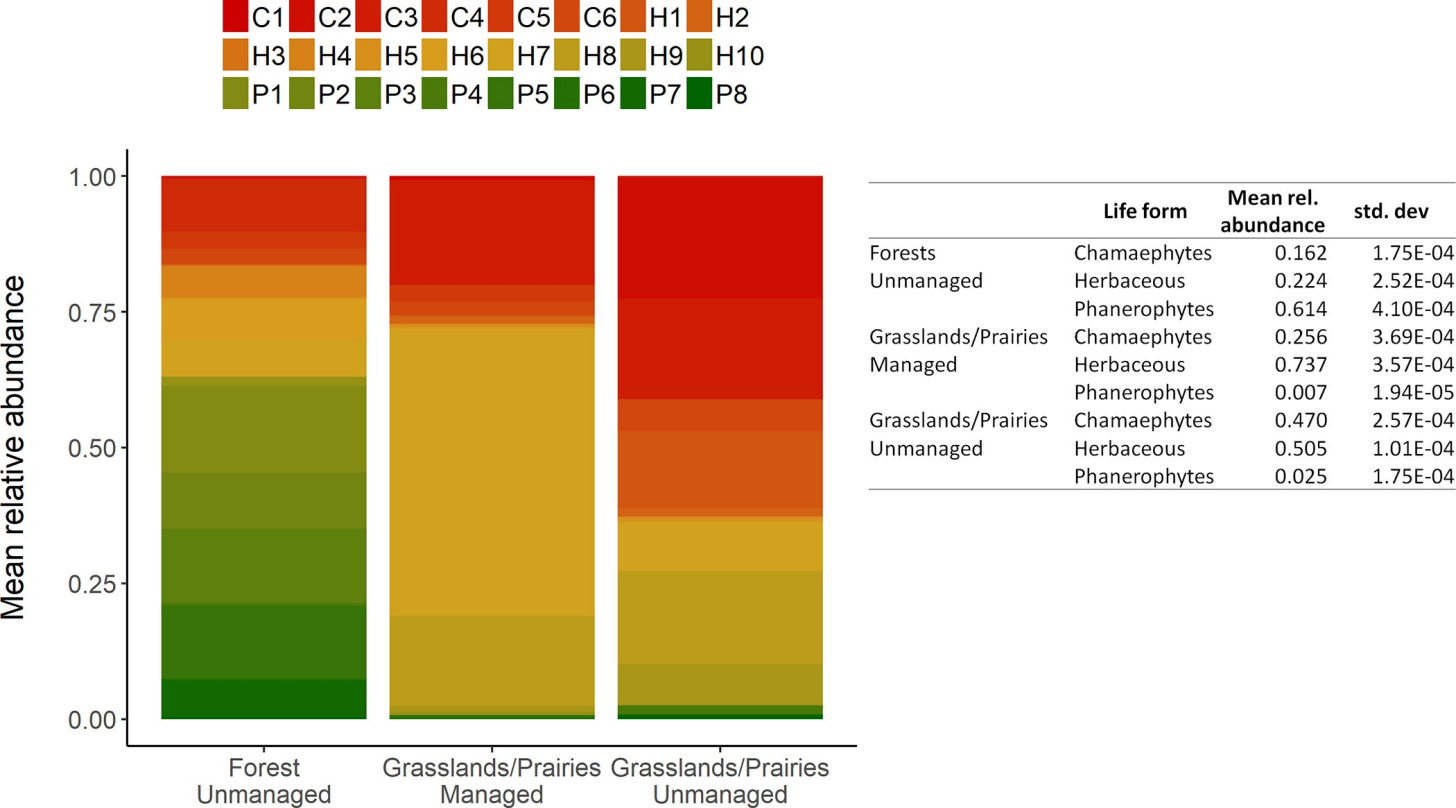
Plant functional group (PFG) relative abundances before disturbances. Bars show PFG relative abundances at the end of the initialisation phase, [ed by life form (colour-coded) and averaged by community type (unmanaged forests, managed and unmanaged grasslands) across the three simulation replicates. See Table C in S3 Appendix and Fig A in S4 Appendix for average relative abundances and standard deviations calculated per PFG.

We developed a simulation experiment with three extreme drought scenarios to test how drought influences gradual climate change effects on forests and grasslands under different management practices (Fig C in S4 Appendix). Simulations had three phases: initialisation, scenario and stabilisation. The initialisation phase was necessary to achieve the ‘current’ state of the vegetation (corresponding to the climate average across 1961–1990) and followed the procedure used and validated in Boulangeat *et al*. [25], requiring 850 time-steps to seed PFGs, allow for vegetation succession and mimicking past land use in the Écrins NP. Fig 1 shows the PFG composition by life form in the three communities analysed (unmanaged forests, managed grasslands and unmanaged grasslands) at the end of the initialisation phase, before any disturbances were applied (i.e. gradual climate change and drought).

The scenario phase started from the end of the initialisation, by applying one of three scenarios of drought to the ‘current’ vegetation (150 time-steps representing years 1991–2140): *‘no drought’*, *‘sporadic and moderate drought’* and *‘frequent and severe drought’*. Our choice of drought scenarios was based on a previous fully-factorial experiment [15], from which we selected the most contrasting scenarios to put in evidence drought consequences for ecosystem stability. For the ‘*no drought*’ scenario the current *Din* map (average *Din* between 1961–1990) was fed to FATE-HD and kept until the end of the simulations; for the *‘sporadic and moderate drought’* scenario the moderate *Din* map (projected *Din* for 2080, increased by 20%) was fed to FATE-HD every 16 time-steps (i.e. low drought frequency and intensity); and for the *‘frequent and severe drought’* the severe *Din* map (projected *Din* for 2080, decreased by 20%) was fed to FATE-HD every time-step (i.e. high drought frequency and intensity). Because drought is unlikely to occur every year for decades, we introduced 10 no-drought time-steps between each sequence of 5 drought events. Drought events always started with climate change (time-step 15), but stopped between time-steps 90 and 105, depending on the frequency. All three scenarios included gradual climate change, which was simulated by changing habitat suitability maps between time-steps 15–90 of the scenario phase at every 15 time-steps (equivalent to 2005, 2020, 2035, 2050, 2065 and 2080). Unlike drought, climate warming was kept until the end of the simulations, by keeping the last habitat suitability map. We chose to stop drought while maintaining a warmer climate so that we could investigate how drought impacted the communities over the long term, even in the absence of further drought effects. Thus, after the scenario phase the model ran for an additional 50 time-steps to achieve quasi-equilibrium (stabilisation phase, representing the years 2141–2190) so that long term drought effects could be observed.

All drought simulation scenarios were repeated 3 times, as this adequately captures the range of variation produced by the model [25]. In addition, we ran 100 simulations (scenario and stabilisation phases) without climate change nor drought for null comparisons–‘no change’ simulations. In this case, both current habitat suitability and current *Din* maps were used throughout the simulation (reflecting the 1961–1990 average climate and drought intensity).

### Stability analyses using hypervolumes

We focused our stability analysis on forest (unmanaged) and grassland (managed or unmanaged) communities present in the forest-grassland ecotone (see S2 Appendix for community subsetting procedure). Hypervolumes of PFGs’ yearly relative abundances (averaged across the landscape) were used to represent the state of communities. We compared the current state (i.e. last 45 years of the initialisation phase at which quasi-equilibrium was reached; n = 10) with the future state of communities after different drought scenarios were applied (i.e. 50 years of stabilisation phase; n = 11). For null comparisons, we compared the current state with the future state of communities under ‘no change’ simulations (also using the 50 years of stabilisation phase; n = 11). Departures from average initial PFG abundances were measured as i) between-centroid distances (hereafter, mean distances) of initial and final state hypervolumes. Changes in the temporal variability of PFG abundances were measured ii) as differences in initial and final hypervolume sizes (size changes). Finally, iii) the overlap between initial and final state hypervolumes (overlap) provided an overall measure of community similarity, that compliments the other two metrics [18]. Thus, the more distant centroids become, the larger the final hypervolume is and the less it overlaps with the initial hypervolume, the more unstable the analysed community was with respect to the analysed disturbance.

Before calculating and comparing the hypervolumes, we reduced the number of dimensions to three using Principal Components Analyses (PCA), and chose the ideal bandwidth size using a sensitivity analysis (0.15, see S2 Appendix and [18,41]). For each of the 27 pairs of current and future state hypervolumes (3 community-management combinations x 3 drought scenarios x 3 repetitions), we 1) calculated a PCA on the combined PFG relative abundances of each state; 2) extracted the factor scores from the first three principal components; 3) calculated the current and future states hypervolumes on the factor scores corresponding to these periods; and 4) compared the hypervolumes in terms of mean distance, size changes and overlap. Because hypervolume calculations rely on random sampling techniques, results can be influenced by small sample sizes [41]. To account for this, steps 3 and 4 were repeated 100 times. For null comparisons, each of the 100 pairs of hypervolumes was only compared once, as ‘no change’ simulations were run 100 times.

### Statistical analyses

Since we did not simulate forest management, we did not have a fully crossed design. Hence, we divided our statistical analyses along two main questions: 1) do different drought regimes affect forests and grasslands differently (the effect of habitat)? And 2) do the effects of different drought regimes on grasslands depend on management regime (the effect of management)? To investigate the first question, managed grasslands were excluded from the analysis, and to investigate the second question forests were excluded from the analysis.

The effect of different drought scenarios on hypervolume comparisons was assessed separately for each response variable (mean distance, size changes and overlap), by running analyses of variance (ANOVAs) with and without null comparisons (used as a control treatment). When null comparisons were included, we used Type III ANOVAs to account for the different sample sizes (n = 100 for null comparisons; n = 300 for drought scenario comparisons). Before calculating ANOVAs, we verified normality and homoscedasticity, and log-transformed response variables when necessary to ensure that these conditions were met. For a visual interpretation of results, we calculated the standardised effect sizes (SES) of the different drought scenarios with respect to the null comparisons, per community and management combination.

Finally, we assessed functional changes in forest and grassland communities by fitting yearly community weighted mean (CWM) values of 12 different functional traits (also averaged across the landscape; trait values in Table A in S3 Appendix) to the afore mentioned PCAs, using the function *envfit* in the *vegan R* package. This *post-hoc* approach allowed finding the trait vectors best correlated with axis of the calculated PCAs without constraining the hypervolumes to changes in functional diversity.

## Results

**Fig 2:**
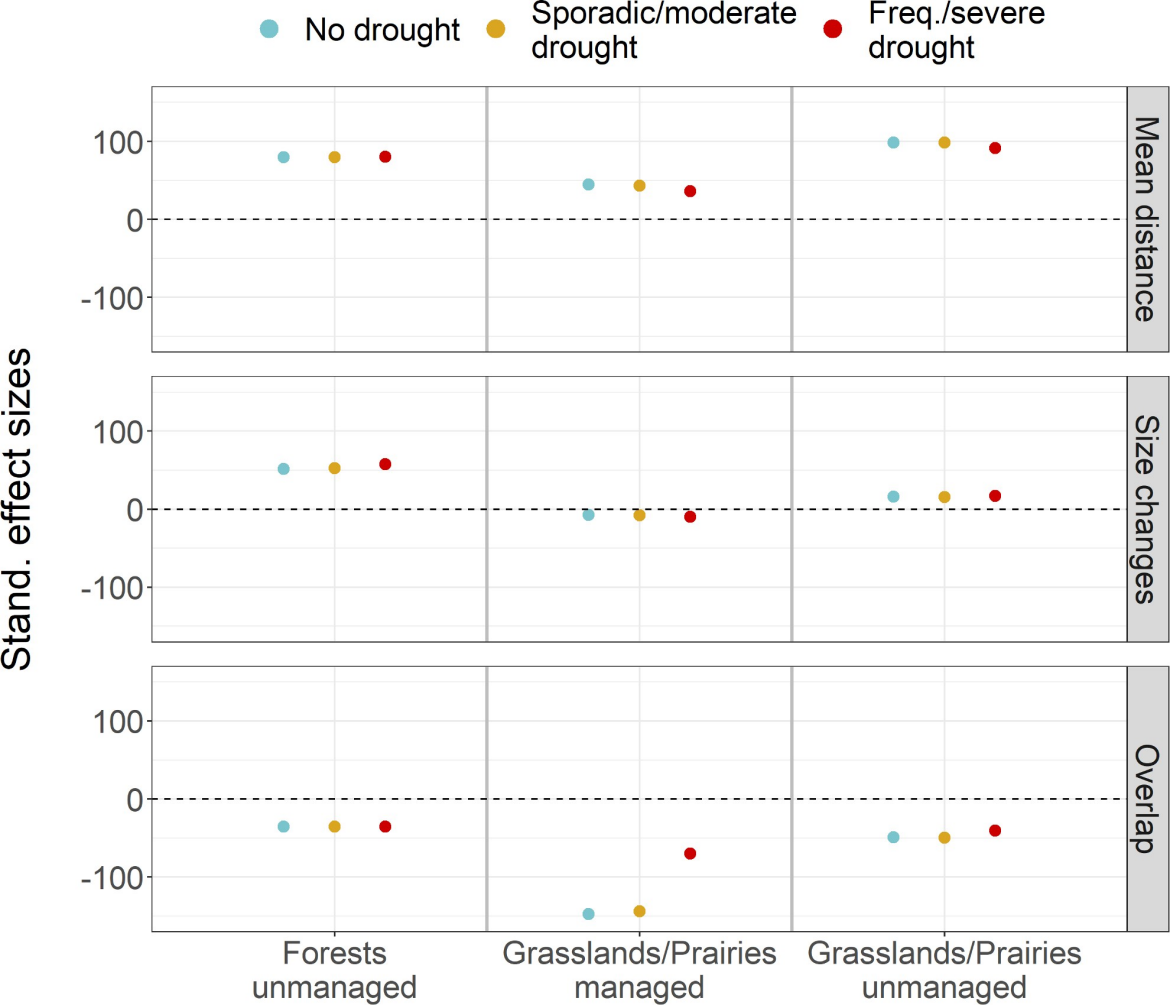
Standardised effect sizes (SESs) of drought scenarios by community and management types on hypervolume metrics, relatively to null comparisons. Dashed lines indicate a zero or no effect. SESs were calculated following Glass’s delta formula, as the mean difference between the scenario and null groups, divided by the standard deviation of the null group [42].

As a first step, we evaluated whether the effect of the drought scenarios and their interactions with type of community and land-use regime were significantly different from random variation, by comparing them to the set of ‘no change’ simulations (i.e. null comparisons). Variance between scenarios was significantly larger than within-scenario variance (Table E in S3 Appendix) and null comparisons had largely different values from drought scenarios (Fig 2). This confirmed that all scenarios significantly affected communities, yet differences between scenarios were less clear. Hence, we tested for the effects of drought scenarios by excluding ‘null comparisons’. Although climate change seemed to be the main driver of community destabilisation (i.e. ‘no drought’ effects were qualitatively similar to the effects of remaining drought scenarios; Fig 2), we still found significant differences between drought scenarios (Table F in S3 Appendix), confirming that different drought scenarios had quantitatively different effects on the future state of communities. Like gradual climate change, drought led to significant changes in the mean and variance of relative PFG abundances (i.e. mean distance and size changes), as well as significant overall changes in community structure. Yet, the impacts of different drought regimes differed between forest and grassland communities, as well as land-use regimes (Fig 2, Table F in S3 Appendix). For instance, frequent and severe drought caused grassland community structure to change less (smaller mean distances and larger overlaps) than other drought scenarios, but it made forests become more variable (larger future hypervolume sizes, Fig 2).

**Fig 3:**
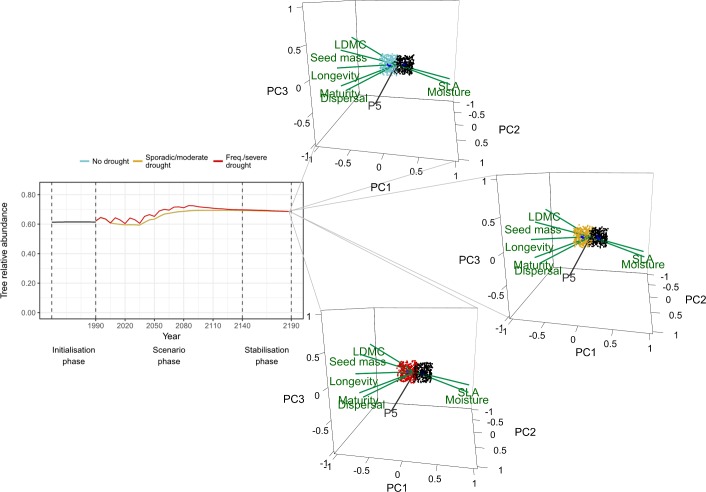
Unmanaged forests. Effects of different drought scenarios on tree transient dynamics and on community states represented by hypervolumes. Current (in black) and future (blue, yellow and red) state hypervolumes are shown with their centroids (in dark blue). Five PFGs with the largest absolute factor loadings on the first three principal components are shown in grey. Functional traits with highest correlations with PC1 (traits with absolute coordinate values ≥ 0.8) are shown in green. Trait vector coordinates were scaled by the corresponding trait vectors’ correlations with ordination axes (only traits with absolute coordinate values ≥ 0.8 on PC1 are shown). For visual clarity, only 300 random sampled points are shown per hypervolume.

**Fig 4:**
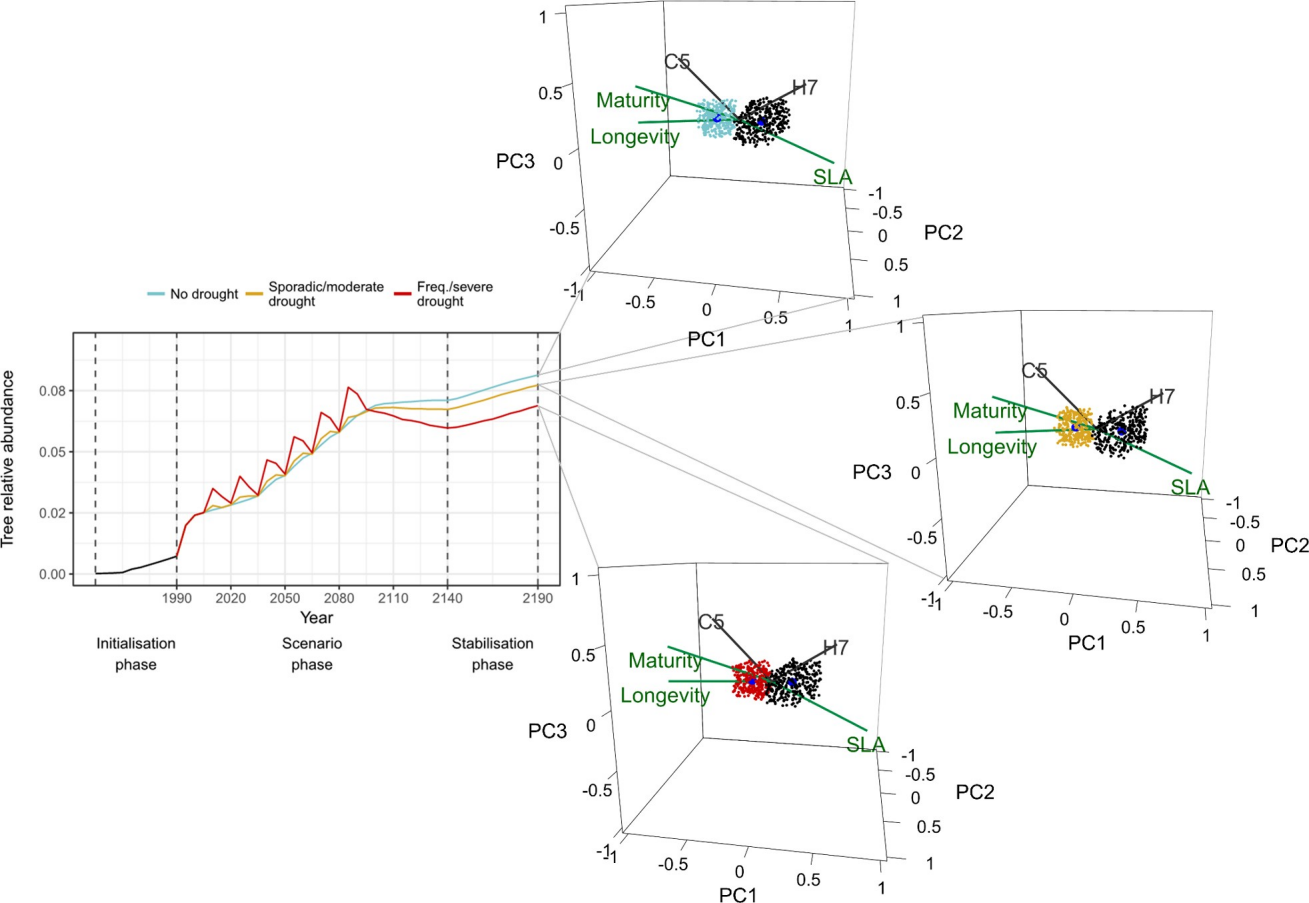
Managed grasslands. Effects of different drought scenarios on tree transient dynamics and on community states represented by hypervolumes. Current (in black) and future (blue, yellow and red) state hypervolumes are shown with their centroids (in dark blue). Five PFGs with the largest absolute factor loadings on the first three principal components are shown in grey. Functional traits with highest correlations with PC1 (traits with absolute coordinate values ≥ 0.8) are shown in green. Trait vector coordinates were scaled by the corresponding trait vectors’ correlations with ordination axes (only traits with absolute coordinate values ≥ 0.8 on PC1 are shown). For visual clarity, only 300 random sampled points are shown per hypervolume.

**Fig 5:**
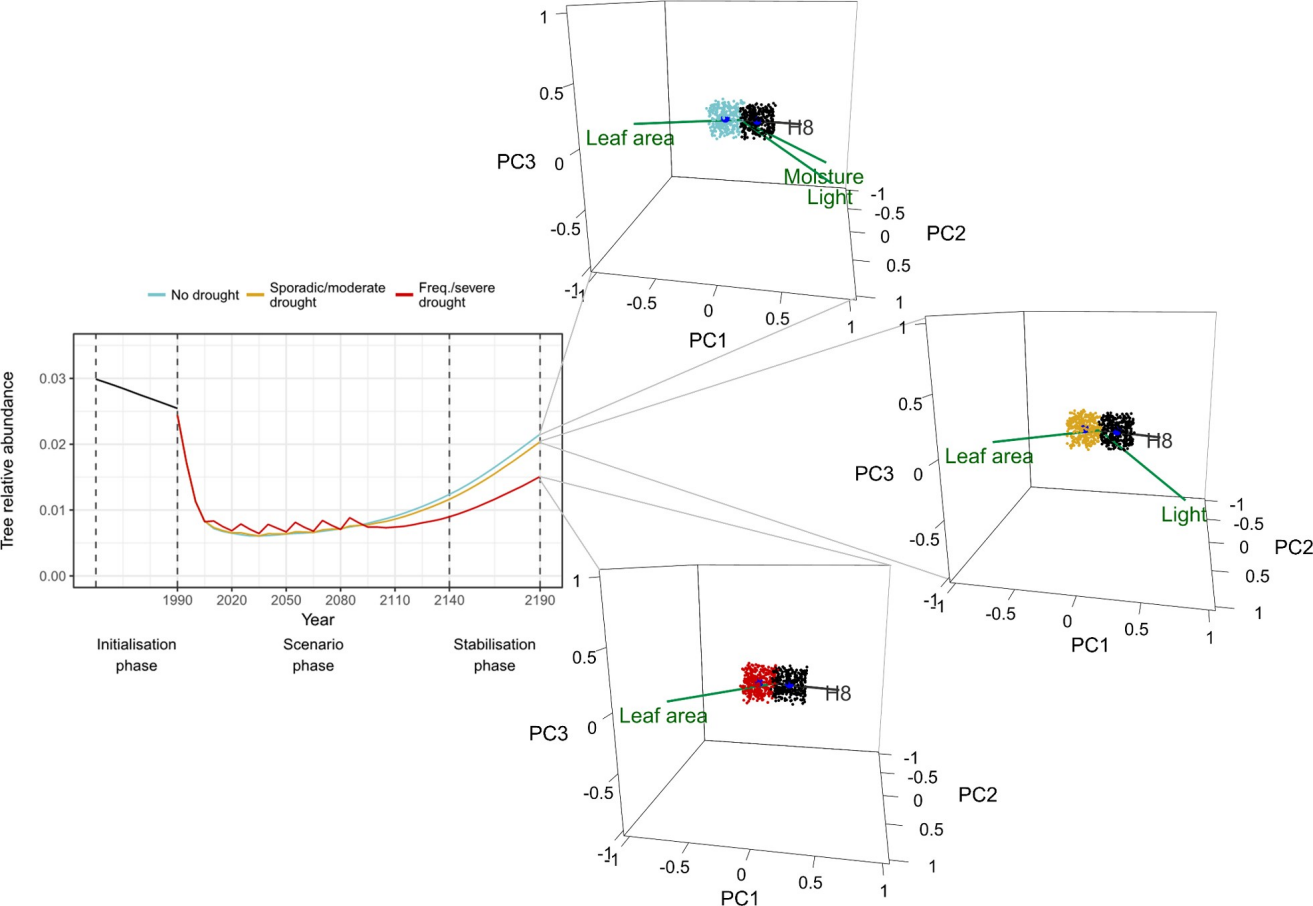
Unmanaged grasslands. Effects of different drought scenarios on tree transient dynamics and on community states represented by hypervolumes. Current (in black) and future (blue, yellow and red) state hypervolumes are shown with their centroids (in dark blue). Five PFGs with the largest absolute factor loadings on the first three principal components are shown in grey. Functional traits with highest correlations with PC1 (traits with absolute coordinate values ≥ 0.8) are shown in green. Trait vector coordinates were scaled by the corresponding trait vectors’ correlations with ordination axes (only traits with absolute coordinate values ≥ 0.8 on PC1 are shown). For visual clarity, only 300 random sampled points are shown per hypervolume.

Notably, the long-term effects of different drought regimes depended on the type of community and land-use regime considered (Figs 2 to 5; Table F in S3 Appendix). Forests were overall more stable under drought and climate change than unmanaged grasslands, having shown smaller departures from mean abundances (i.e. shorter mean distances) and changing less in terms of overall community structure (i.e. larger overlaps; Fig 2), despite abundances becoming more variable in the future. Although differences between drought regimes were small, frequent and severe drought led to slightly more variable forests in the future (higher increases in size; Fig 2). Being more stable, forests also showed relatively weak taxonomic changes when compared to grasslands, as all PFG eigenvector values were <0.5 (Fig 3, Fig H in S4 Appendix). Nonetheless, riparian pioneer trees (P2), late successional deciduous trees (P3) and undergrowth groups (H4, H6 and H7) decreased in abundance, while thermophilous pioneers (P1), late successional trees (P5, P7), and drought tolerant shrubs and woody chamaephytes (C4 and C5, respectively) increased. This caused several functional changes at the community level. Forests suffered reductions in average specific leaf area (SLA) and community moisture requirements, while traits like dispersal distance, longevity, seed mass, maturity and leaf dry matter content (LDMC) increased. Interestingly, the added effect of drought did not impact taxonomic or functional changes already observed under gradual climate change (Fig 3; Fig H in S4 Appendix).

Managed grasslands were also more stable to drought and climate change than their unmanaged counterparts, showing smaller departures from initial mean PFG abundances and varying less in the future, even if they appeared to change more in terms of community structure (i.e. smaller overlaps; Fig 2). In fact, smaller overlaps were likely caused by the considerable size reductions in future state hypervolumes (compare Figs 4 and 5). Like in forests, changes in managed grasslands were qualitatively similar across drought scenarios, yet, quantitatively, frequent and severe drought led to smaller overall departures from the current state (note the smaller mean distances and larger overlaps, also present in unmanaged grasslands; Fig 2). Taxonomic changes in managed grasslands, however, were similar across drought scenarios, being mostly driven by non-palatable and light-loving PFGs that are relatively abundant in these communities (see Tables A and F in S3 Appendix, and Fig I in S4 Appendix). Undergrowth (H7) and alpine to subalpine (H8) herbaceous groups were replaced by semi-woody and dispersal limited chamaephytes (C3, containing woody shrubs like *Rhododendron ferrugineum*, *Salix herbacea*, *Salix reticulata*, and *Salix retusa*) and mountainous to subalpine drought-tolerant heath (C5, composed of *Arctostaphylos uva-ursi crassifolius*, *Calluna vulgaris*, *Hippocrepis emerus*). This led to reductions in community average SLA and increased overall longevity and maturity (Fig 4).

Unmanaged grasslands suffered mostly from the loss of groups characteristic of alpine environments, with subalpine and alpine herbaceous groups (like H1, H8 and H9) being replaced by more thermophilous and drought tolerant chamaephytes (C1) and herbs (H5; see Tables A and F in S3 Appendix, Fig J in S4 Appendix). Unlike in forests and managed grasslands, in unmanaged grasslands there were distinct impacts of drought scenarios on functional structure. In agreement with results shown by hypervolume metrics, frequent and severe drought caused smaller changes in the functional structure of unmanaged grasslands, mostly increasing community-averaged leaf area. Sporadic and moderate drought caused, in addition, decreases in light preference, while gradual climate change also led to decreases in moisture requirements (Fig 5).

## Discussion

Climate change and extreme drought events will affect the stability of forest-grassland ecotone communities in the European Alps, even if land use remains unchanged. Gradual climate change was the main driver of long-term destabilisation of grassland and forest communities. Nevertheless, drought had strong short-term effects on communities, and its impacts on their long-term structure and composition depended on the type of community and land-use regime. For instance, frequent and severe drought offered a short-term advantage to woody PFGs in managed grasslands, but led them to be less encroached in the long term (Fig 4; see also [15]). Also, drought impacts on structural stability showed that forests were generally more stable than grasslands. Indeed, turnover of forest species (undergrowth or canopy) was more limited by biotic filtering, as new species needed to be shade tolerant. Slower phanerophyte dynamics (they grow slower, live longer and mature later) and their higher drought tolerances also contributed to forest stability, especially because established canopies reduced drought intensity and protected communities from extreme drought effects. Nevertheless, we still observed a shift to more drought tolerant forests. For instance, the more positive long-term effect of drought on late-successional and shade-tolerant PFGs like *Picea abies* (in P5) relatively to more thermophilous pioneer trees like *Pinus sylvestris* (in P1) agrees with field studies. In Valais, Switzerland, *P*. *abies* has been shown to be facilitated by drought, invading *P*. *sylvestris* stands and outcompeting the later species over the long term [31]. Our results also concur with studies indicating that changes in forest composition will also occur at the undergrowth level [5] with the loss of moisture-loving undergrowth species.

Despite there being more woody encroachment in managed grasslands (note the increase in tree relative abundance in Fig 4), they also appeared to be more stable than unmanaged grasslands, as mean PFG abundances changed less and became less variable. This was likely due to two things. First, grazing limited the species that could colonise managed grasslands, preventing large turnovers, but allowing an increase of young trees and seedlings that caused some woody encroachment. Second, unmanaged grasslands are situated at higher elevations (2736 m a.s.l. on average, across repetitions) where changes in abiotic conditions can easily destabilise communities by driving turnover towards more drought tolerant and warm-adapted species [11]. In fact, the loss of subalpine and alpine grassland species (like groups H1, H8 and H9) and the increases in the woody/non-woody ratio that we observed in grasslands have been indicated as impacts of climate change in field studies [14,43]. These shifts caused functional changes in grasslands, which may implicate lower total productivity, and lower fodder quantity and quality [44]. For instance, experimental studies in subalpine grasslands also reported decreases in average SLA with drought, together with lower nutritive value of forage [45]. In this case, functional changes were attributed to phenotypic plasticity, rather than turnover, and communities rapidly recovered after drought (see also [46]). Admittedly, FATE-HD does not reproduce intra-PFG variability; yet, prolonged and repeated drought events may degrade community recovery potential and cause longer-term changes, like the observed turnover, that are not visible during short-term studies [47].

While past studies looking at drought effects focused on the stability of particular ecosystem functions [16,33,48] and individual community properties [15,47,49], we focused on multidimensional structural stability [50]. This way, we analysed community disturbances holistically, which not only is a simpler analysis from a methodological point of view–analysing changes many different community properties was not necessary–but is also a better representation of how biodiversity responds to disturbances. We recommend that future studies looking at how ecosystems respond to disturbances also consider changes in structural stability using multidimensional approaches like the hypervolumes framework, rather than following single properties that may not respond disturbance and falsely indicate the absence of change [18,51]. Importantly, the hypervolumes framework allows for cross-community and cross-scenario comparisons, which can be important in from a management perspective as limited resources often mean prioritising more sensitive communities for conservation efforts. Although here we used a simulation experiment, the framework can be applied to empirical data and to analyse changes in other ecosystem, or community components [18]. For instance, space-for-time analyses can be done using treatment-type field experiments, provided that enough replicates are available in each treatment. The hypervolumes framework also allowed readily analysing which community entities (in this case PFGs) were most responsive to drought scenarios, and how this reverberated to changes in functional diversity. Because community functional trait values are tightly linked to ecosystem services [8], our results are thus highly relevant for ecosystem management in this region. At present, much of the management in the Écrins NP, and elsewhere in the European Alps, aims to prevent the loss of open habitats and associated biodiversity and ecosystem services [10,52] by subsidising traditional pastoral activities and preventing woody encroachment [53,54]. Yet, shifts in forest structure and composition can have important repercussions for general biodiversity, and affect carbon and water cycles [4,55]. For instance, the ‘eucalyptus dieback’ in Australia has been followed by sharp declines of avian fauna [56]. Forest dieback may also affect the carbon balance by decreasing carbon uptake through foliage and increase carbon emissions from stemwood decomposition (see review by [57]). Although we have not included forest management in our study, we can foresee that the combined impact of management, drought and gradual climate change on forest stability will largely depend on the type of management in terms of structural and composition diversity (e.g. lower overstory densities can decrease competition for water [58] species mixtures can increase resilience to drought [59]). Managing for high diversity of ecosystem services in forest-grassland ecotones will therefore require an assessment of the relative stability of grasslands and forests to global change drivers. This means that we need to understand impacts on both taxonomic and functional diversity, so that more resilient community structures and compositions can be promoted.

Finally, as in any other model, our results are linked to how climate change and drought were parameterised. The fact that gradual climate change drove the long-term dynamics of community structure is linked to climate change effects being kept until the end of the simulation, while drought events ended before the stabilisation phase. This enabled testing whether drought effects (on top of climate change) would be long lasting, even after ceasing drought events. Even if our results cannot be verified on the long term, we defend that designing best- and worst-case scenarios, as we did here, aids our understanding of how drought may impact ecosystem stability in the future [60]. Furthermore, the results obtained using our model and parameterisation agreed with those obtained by others in field studies. Hence, although lack of data prevented us from using a trait-based approach to drought simulation, we trust that our statistical approach reflects the general drought tolerance of these PFGs. Especially considering that PFG responses to drought were parametrised and validated in collaboration with botanists working within the study area. Finally, other drivers, such as carbon, nutrient and water cycles and pest outbreaks are known to interact with drought in affecting vegetation dynamics [55,61]. We could expect stronger drought effects if these factors were to be included in our model; yet, unfortunately, we do not currently have the data that would enable us to simulate these processes at large spatial scales and across multiple plant groups. Existing dynamic vegetation models, like LANDIS-II, have been coupled to carbon, nutrient and water cycle models (see e.g. [62,63]), but also insect outbreak dynamics [64]. These models focus on forest stand dynamics and future work is needed to expand them to other ecosystems, as responses can vary across vegetation types [65]. Doing so requires systematic quantification how different plant groups respond to and affect these bio-geochemical cycles and pest dynamics, which is still lacking [65].

To conclude, our simulation study showed that drought may not reverse on-going impacts of gradual climate change in forest-grassland ecotones. Still, its impacts on final community structure will likely differ between forest and grasslands, as well as land-use regimes, and may impact the provisioning of ecosystem services in the European Alps. Notably, the hypervolumes framework allowed a comprehensive analysis of the impacts of distinct disturbances for the structural stability of distinct plant communities, with a direct interpretation of what they meant for biodiversity and, consequently, ecosystem services.

## Supporting information

S1 Appendix The FATE-HD simulation platform and drought simulation experiment. (DOCX)

S2 Appendix Applying the hypervolumes framework and statistical results. (DOCX)

S3 Appendix Supplementary tables. (DOCX)

S4 Appendix Supplementary figures. (DOCX)
