# Supplementary material for: Drought effects on the stability of forest-grassland ecotones under gradual climate change
Source: PLoS One. 2018 Oct 24;13(10):e0206138. doi: 10.1371/journal.pone.0206138 (PMC6200273; doi:10.1371/journal.pone.0206138)
Supplement: S1 Appendix — (DOCX) [file pone.0206138.s001.docx]

**S1 Appendix**

**The FATE-HD simulation platform and drought simulation experiment**

*Climate data for habitat suitability maps*

Habitat suitability maps were calculated for each PFG based on seven environmental variables (slope, percentage of calcareous soil and five bioclimatic variables). Slope angle was obtained from the French Digital Elevation Model with 50x50m resolution, made by the IGN-France (<http://professionnels.ign.fr/bdalti>). Percentage of calcareous soil was calculated from the European Soil Database (<http://eusoils.jrc.ec.europa.eu/data.html>) at 1 km resolution as the percentage of the area of Soil Typological Units (STU) with calcareous dominant parent material for every Soil Mapping Unit (SMU) within the Alpine range [see also 1]. For ‘current’ habitat suitability values of the five bioclimatic variables (isothermality, temperature seasonality, temperature annual range, mean temperature of coldest quarter, and annual precipitation) were obtained from WordClim (http://www.worldclim.org/) for the period of 1961-1990, and downscaled to 100 x 100 m resolution using the change factor method [2]. ‘Future’ habitat suitability was based on climate projections that followed IPCC previsions of the A1B scenario for years 2020, 2050 and 2080. These were obtained using the regional climate model (RCM) RCA3 [3] fed by the global circulation model (GCM) CCSM3 (derived from the ENSEMBLES EU project outputs [4]).

*Calculating habitat suitability*

Habitat suitability maps were calculated as in Boulangeat *et al.* [5] and will only be briefly described here.

Habitat suitability models were run for each PFG using a species distribution modelling approach. PFG occurrences were compiled from occurrence data available for their representative species from the Conservatoire Botanique National Alpin (CBNA) vegetation-plot database, covering most of the French Alps [6,7]. Only data from exhaustive *relevés* (identification of all plant species within a plot) from after 1980 were used (ca. 15,000 plots). A PFG was considered present in a given plot if at least one of its representative species was observed. Seven environmental variables (slope, percentage of calcareous soil, isothermality, temperature seasonality, temperature annual range, mean temperature of coldest quarter and annual precipitation – described above) were used as predictors. For ‘current’ habitat suitability, bioclimatic variables were averaged across 1961-1990. For ‘future’ habitat suitability, we used projections of the same bioclimatic variables for 2020, 2050 and 2080.

Five different statistical models were used to describe the relationship between PFG presence/absence and environmental predictors: Generalized Linear Model, Boosted Regression Trees, Generalized Additive Model, Multivariate Adaptive Regression Splines and Random Forest. Presences and absences were equality weighted, to ensure comparability between PFGs. Each model was calibrated across the whole French Alps using a random sample of 70% of the available data, the remaining 30% being kept for evaluation with True Skill Statistics (TSS; [8]). Calibration and validation were repeated 10 times for each model and each PFG. Finally, ensemble forecasts were obtained for each PFG to derive their probabilities of occurrence (i.e. habitat suitability). For this, all statistical models were used and their probabilities of occurrence were first binarized using the threshold that maximised TSS in their evaluation. Model binary projections were then weighted according to their TSS scored and summed. Finally, weighted sums were rescaled between 0 (low) to 1 (high) to provide the habitat suitability value of any given pixel for a PFG.

*Climate data for drought intensity maps*

Climate data used to calculate ‘current’ drought intensity (*Din*) maps was obtained from projections of the meteorological model Aurelhy [9] for the 1961-1990 period, and interpolated at 100 m resolution across the whole French Alps. ‘Future’ *Din* maps were calculated using climate variable projections for 2080 obtained using the same modelling approach as described above for BIOCLIM variables.

*Moisture index (*MI*) and drought intensity (*Din*) calculations*

Monthly moisture index (*MI*) values were calculated for each 100 x 100m pixel across the entire French Alps as:

$$MI_{i}=\sum_{j=1}^{n} P_{j}-PET_{i}\times n+0.5$$

with *Pj* being daily precipitation, *PETi* being the average daily potential evapotranspiration of month *i* – calculated following Turc’s [10] formula – and *n* the number of days in month *i*. Drought intensity (*Din*) values were calculated per pixel as the lowest monthly value of *MI* observed in each year. Hence, the more negative a *Din* value was, the more severe drought was in a given year.

*PFG historical* Din *distributions and soil moisture requirements*

Historical *Din* distributions between 1961-1990 (*Din_1961-1990_*) were calculated for each PFG and used to define PFG-specific drought thresholds (see main text). To do so, we extracted yearly *Din* values from all pixels across the entire French Alps where the PFG was present – i.e. at least one of its representative species was recorded (for species list see Table B in S3 Appendix). See above in *Habitat suitability maps* for details on the occurrence data used.

To calculate a PFG’s soil moisture requirements, we assumed that the historical distribution of *MI* values recorded where a PFG occurs reflect its moisture requirements and adaptations to drought. Hence, we extracted PFG-specific *MI* distributions between 1961-1990 (*MI_1961-1990_*) following procedure explained above for *Din_1961-1990_*. We then calculated x̅ - 2.5 SD of *MI_1961-1990_* for each PFG and scaled the results into four classes from 0 (low moisture requirements) to 3 (high moisture requirements). These classes were then adjusted according to expert knowledge of the soil moisture preferences of the species present in the PFGs, resulting into four final classes ranging from 0-3 (Table A in S3 Appendix).

*Validation of drought parametrisation*

The parametrisation of drought-related parameters is available in Barros *et al.* [11] is only briefly revisited here (see [11] for further details). Barros *et al.* [11] have simulated historical drought between 1961-1990 and assessed whether the final vegetation state corresponded to what was recorded in the DELPHINE database [12], which describes vegetation composition and structure for 18 381 plots covering the whole park.

The validation simulation was started for the 800^th^ time-step of the initialisation phase, after which maps of observed yearly *Din* values were fed to FATE-HD for 30 time-steps (see above for *Din* calculation). Since the parametrisation of PFG responses was based on the same climatic period, vegetation composition and structure were expected to remain fairly similar to what had been achieved by Boulangeat *et al.* [5] and recorded in the DELPHINE database. Several combinations of parameter values were tested before the final validation presented here. Throughout these trials, parameter values were discussed with expert botanists working on the Écrins NP to make sure that they stayed within sensible ranges. Before model accuracy was finally tested for a final set of parameter values, expert botanists visually judged whether PFG distributions produced by parameter value combinations were realistic.

To validate vegetation composition, PFG presences and absences obtained at the end of the validation simulation were compared to DELPHINE data, after converting the 90 DELPHINE vegetation types to presences/absences of our 24 PFG (see [5] for details). Model specificity (proportion of correctly predicted PFG presences – true positives), model sensitivity (proportion of correctly predicted PFG absences – true negatives) and error rate (overall proportion of false positives and false negatives) were calculated for each PFG, and resulting values were compared to the specificity, sensitivity and error rate of the PFG habitat suitability models. Here, we also added the results obtained by Boulangeat *et al.* [5] for an easier comparison to the base model.

To validate vegetation structure, simulated tree cover and strata abundances were visually compared to presence/absence data of three vegetation strata (herbaceous, woody with <4m and woody with >4m; DELPHINE database). This was done separately for different habitats considering tree cover only, and across the landscape for each of DELPHINE’s three height levels. In both cases results obtained with drought were also compared with results obtained by Boulangeat *et al.* [5].

The predicted accuracy (error rate) of PFG distributions was very similar to that of the base model [5], with slight increases for seven PFGs and decreases for six PFGs (Table D in S3 Appendix). Simulating past drought leads to lower abundances across PFGs (data not shown). It also improves general estimates of tree cover in rocky and alpine habitats, but leads to underestimated tree cover in pasture fields, lowlands and mountainous forests. In subalpine and mountainous open habitats tree cover, which was previously overestimated in the base model [5], is now underestimated, yet it departs less from the observed cover in absolute terms (Fig D in S4 Appendix). In general, simulated strata abundances remain consistent with observed presences and absences, as larger strata are predicted to be more abundant where they are observed (Fig E in S4 Appendix).
