# Supplementary material for: Drought effects on the stability of forest-grassland ecotones under gradual climate change
Source: PLoS One. 2018 Oct 24;13(10):e0206138. doi: 10.1371/journal.pone.0206138 (PMC6200273; doi:10.1371/journal.pone.0206138)
Supplement: S2 Appendix — (DOCX) [file pone.0206138.s002.docx]

**S2 Appendix**

**Applying the hypervolumes framework and statistical results**

*Preparing simulation outputs for hypervolume analysis*

We explored the effects of gradual climate change and different drought regimes on the stability of forest and grassland communities situated on the forest-grassland ecotone belt. The ecotone belt was spatially delimited using the first year of one *no drought* scenario repetition (as this year was similar across all simulations) and fixed across remaining years to follow the temporal dynamics of the same ecotone communities (i.e. pixels). The ecotone was delineated as buffer drawn 500 m below and 1000 m above the upper tree line. The upper tree line was defined at the third quartile of elevation values of pixels with > 60% tree cover (i.e. phanerophyte plant functional groups with > 1.5 m). We subset forest and grassland communities based on tree cover (as defined above). Following forest and grassland habitat classifications in DELPHINE [12], pixels with > 60% tree cover were classified as forests, while pixels with < 10% tree cover and > 60% cover of the herbaceous stratum (< 1.5m) were classified as grasslands. We then selected forest and grassland pixels that had no grazing or mowing activities (unmanaged forests and unmanaged grasslands), and grassland pixels that were subjected to intermediate grazing levels (managed grasslands).

Raw PFG abundances were averaged across all pixels of the same community-management combination, per year, before calculating relative yearly PFG abundances. There were cases where average raw abundances were zero for a given PFG across a whole period of analysis (current or future). So to avoid suppressing PFGs (and thus changes in dimensionality) we added 0.000001 to the missing groups before calculating the relative abundances.

*Dimensionality reduction*

It is recommended that hypervolumes are built from a maximum of 5-8 dimensions to avoid having highly disjunct hypervolumes (i.e. hypervolumes with “holes” [13]). Thus, we reduced the number of dimensions from the initial 24 PFG abundance variables following the approach detailed in Barros et al. [14]. We calculated Principal Components Analyses (PCAs) for each pair of compared states – i.e. on the joint datasets of yearly PFG relative abundances from the current and future state periods – and extracted the factor scores from the first 3 PCA axes, at which the cumulative explained variance saturated close to 1.0 (Fig F in S4 Appendix). This approach also ensured that the axes used to calculate hypervolumes were orthogonal. The unusually high proportion on explained variance stems from the fact that the changes in PFG composition caused simulated disturbances are almost totally captured by the first axes. We nevertheless chose to proceed using 3 axes so that we could investigate changes in hypervolume sizes.

*Bandwidth selection and sensitivity analysis*

Bandwidth sizes should be large enough to avoid disjunct hypervolumes [13], hence we ran a sensitivity analysis to assess how varying bandwidths affected disjunct factor values and resulting hypervolume comparisons.

We chose the interval to vary bandwidths based on initial bandwidth estimates calculated on the factor scores of each PCA (note that one PCA was calculated per current vs. future comparison). Initial bandwidths were calculated on PCA factor scores separately for each axis, using two methods: the Silverman bandwidth estimator [13], and standard deviations. The maximum obtained value across all PCAs (≈ 0.15) determined the magnitude and mid-value of the bandwidth gradient for the sensitivity analysis (30 bandwidth values equally spaced between 0.01 and 0.3). Current and future hypervolume pairs (all 27 pairs were used – 3 scenarios x 3 repetitions x 3 community-management combinations), together with null comparison hypervolume pairs (only 3 were used) were tested for the entire range of bandwidths. For the sensitivity analysis, hypervolume calculations and comparisons were repeated 10 times for each bandwidth value.

Disjunct factor values stabilised close to 0.1 around bandwidths of 0.15 (well below the maximum recommended value of 0.9; [13]; Fig Ga in S4 Appendix). Hypervolumes did not intersect (overlap = 0) for bandwidths smaller than 0.12 and, as expected, the proportion of overlap increased with increasing bandwidth (because hypervolumes become larger). Mean distances between hypervolume centroids were relatively stable across bandwidth sizes, while size changes, albeit quite small, were negatively related with bandwidth size (Fig Gb in S4 Appendix).

As for the qualitative results of drought scenarios, their relative effects on mean distance, size changes and overlap were similar across bandwidth values. Null comparisons always resulted in smaller mean distances, smaller size changes and larger overlaps, relatively to drought scenarios. Frequent and severe drought also led to smaller mean distances and larger overlaps relatively to other drought scenarios (Fig Gb in S4 Appendix). Hence, we chose a final bandwidth value of 0.15, which was close to stabilisation point of disjunct factor values and to the minimum bandwidth value that allowed overlaps between the current and future state hypervolumes.

**References**

1. Dullinger S, Gattringer A, Thuiller W, Moser D, Zimmermann NE, Guisan A, et al. Extinction debt of high-mountain plants under twenty-first-century climate change. Nat Clim Chang. Nature Publishing Group; 2012;2: 619–622. doi:10.1038/nclimate1514

2. Diaz-Nieto J, Wilby RL. A comparison of statistical downscaling and climate change factor methods: impacts on low flows in the River Thames, United Kingdom. Clim Change. 2005;69: 245–268. doi:10.1007/s10584-005-1157-6

3. Samuelsson P, Jones CG, Willén U, Ullerstig A, Gollvik S, Hansson U, et al. The Rossby Centre Regional Climate model RCA3: model description and performance. Tellus A. 2011;63: 4–23. doi:10.1111/j.1600-0870.2010.00478.x

4. NCAR community. Community Climate System Model, version 3.0. [Internet]. June 2004. 2004. Available: http://www.cesm.ucar.edu/models/ccsm3.0

5. Boulangeat I, Georges D, Thuiller W. FATE-HD: a spatially and temporally explicit integrated model for predicting vegetation structure and diversity at regional scale. Glob Chang Biol. 2014;20: 2368–2378. doi:10.1111/gcb.12466

6. Boulangeat I, Philippe P, Abdulhak S, Douzet R, Garraud L, Lavergne SSS, et al. Improving plant functional groups for dynamic models of biodiversity: at the crossroads between functional and community ecology. Glob Chang Biol. 2012;18: 3464–3475. doi:10.1111/j.1365-2486.2012.02783.x

7. CBNA. Conservatoire Botanique Nationale Alpin [Internet]. Segura P, editor. 2015 [cited 1 Sep 2015]. Available: http://www.cbn-alpin.fr

8. Allouche O, Tsoar A, Kadmon R. Assessing the accuracy of species distribution models: Prevalence, kappa and the true skill statistic (TSS). J Appl Ecol. 2006;43: 1223–1232. doi:10.1111/j.1365-2664.2006.01214.x

9. Benichou P, Le Breton O. Prise en compte de la topographie pour la cartographie des champs pluviometriques statistiques. Une application de la methode Aurelhy: la cartographie nationale de champs de normales pluviometriques. Météorologie. 1987;

10. Turc L. Evaluation des besoins en eau d’irrigation, évapotranspiration potentielle. Ann Agron. 1961;12: 13–49.

11. Barros C, Guéguen M, Douzet R, Carboni M, Boulangeat I, Zimmermann NE, et al. Extreme climate events counteract the effects of climate and land-use changes in Alpine tree lines. Mori A, editor. J Appl Ecol. Dryad Digital Repository; 2017;54: 39–50. doi:10.1111/1365-2664.12742

12. Esterni M, Rovera G, Bonet R, Salomez P, Cortot H, Guilloux J. DELPHINE - Découpage de l’Espace en Liaison avec les Potentialités Humaines et en Interrelation avec la Nature [Internet]. 2006. Available: http://www.ecrins-parcnational.fr/sites/ecrins-parcnational.com/files/fiche_doc/12083/2006-atlas-delphine.pdf

13. Blonder B, Lamanna C, Violle C, Enquist BJ. The n‐dimensional hypervolume. Glob Ecol Biogeogr. 2014;23: 595–609. doi:10.1111/geb.12146

14. Barros C, Thuiller W, Georges D, Boulangeat I, Münkemüller T. N- dimensional hypervolumes to study stability of complex ecosystems. Bellwood D, editor. Ecol Lett. 2016;19: 729–742. doi:10.1111/ele.12617

15. Kattge J, Díaz S, Lavorel S, Prentice IC, Leadley P, Bönisch G, et al. TRY - a global database of plant traits. Glob Chang Biol. 2011;17: 2905–2935.
