# Supplementary material for: Drought effects on the stability of forest-grassland ecotones under gradual climate change
Source: PLoS One. 2018 Oct 24;13(10):e0206138. doi: 10.1371/journal.pone.0206138 (PMC6200273; doi:10.1371/journal.pone.0206138)
Supplement: S3 Appendix — (DOCX) [file pone.0206138.s003.docx]

**S3 Appendix - Supplementary tables**

**Table A.** Trait values of the simulated plant functional groups’ (PFGs). Groups belong to one of three life form classes: chamaephytes (C1-6), herbaceous (H1-10), or phanerophytes (P1-8). PFGs with larger values of ‘light’, ‘dispersal’ and ‘palatability’ are, respectively, light-loving, long-distance dispersers and more palatable. ‘No. strata’ indicates the maximum stratum that a PFG can reach (1: 0-1.5 m; 2: 1.5-4 m; 3: 4-10 m; 4: 10-20 m; 5: >20 m). ‘SLA’ and ‘LDMC’ stand for average specific leaf area and average leaf dry matter content, respectively. SLA values for species of PFGs H10 and P8 were obtained from Kattge *et al.* [15]. All PFG trait values are averaged across species – in the case of soil moisture requirements (‘soil moisture’) *MI* distributions were built using occurrence data of PFGs’ representative species (see above for details). Table partially adapted from Boulangeat *et al.* [6] and Boulangeat *et al.* [5], and identical to table in Barros *et al.* [11].

| PFG | No.  strata | Dispersal | Light | Height  (cm) | Palatability | Longevity  (years) | Maturity  (years) | Seed mass  (g) | SLA  (mm^2^ mg^-1^) | LDMC  (mg g^-1^) | Leaf area  (mm^2^) | Soil moisture |
| --- | --- | --- | --- | --- | --- | --- | --- | --- | --- | --- | --- | --- |
| C1 | 1 | 6 | 7 | 27 | 3 | 27 | 5 | 23.91 | 19.21 | 262.74 | 12.95 | 0 |
| C2 | 1 | 4 | 8 | 13 | 3 | 19 | 4 | 0.38 | 18.02 | 196.03 | 1.05 | 2 |
| C3 | 1 | 1 | 8 | 7 | 0 | 45 | 6 | 0.51 | 14.39 | 221.21 | 0.66 | 2 |
| C4 | 2 | 6 | 6 | 209 | 2 | 158 | 10 | 192.99 | 16.83 | 330.52 | 16.97 | 1 |
| C5 | 1 | 6 | 6 | 76 | 0 | 39 | 8 | 75.01 | 8.28 | 390.18 | 0.94 | 0 |
| C6 | 1 | 7 | 6 | 18 | 2 | 92 | 8 | 39.50 | 13.40 | 354.97 | 0.86 | 2 |
| H1 | 1 | 3 | 8 | 17 | 3 | 11 | 4 | 0.86 | 17.22 | 260.65 | 5.00 | 2 |
| H2 | 1 | 6 | 7 | 42 | 3 | 10 | 3 | 4.04 | 22.11 | 250.74 | 18.76 | 2 |
| H3 | 1 | 7 | 7 | 50 | 3 | 9 | 3 | 2.37 | 24.43 | 238.24 | 79.05 | 2 |
| H4 | 1 | 3 | 5 | 76 | 0 | 7 | 4 | 0.36 | 29.76 | 228.53 | 541.13 | 2 |
| H5 | 1 | 3 | 7 | 40 | 3 | 7 | 4 | 1.94 | 20.71 | 243.02 | 31.34 | 1 |
| H6 | 1 | 3 | 6 | 73 | 3 | 8 | 4 | 2.31 | 28.21 | 227.85 | 76.68 | 2 |
| H7 | 1 | 5 | 6 | 19 | 0 | 7 | 4 | 0.40 | 19.25 | 195.45 | 97.07 | 2 |
| H8 | 1 | 3 | 8 | 19 | 0 | 8 | 4 | 0.89 | 23.11 | 274.24 | 0.18 | 3 |
| H9 | 1 | 7 | 8 | 19 | 3 | 9 | 4 | 0.38 | 21.09 | 417.58 | 1.40 | 3 |
| H10 | 1 | 7 | 6 | 100 | 3 | 9 | 4 | 6.20 | 21.14 | 0.22 | 353.31 | 2 |
| P1 | 3 | 6 | 6 | 1175 | 2 | 193 | 15 | 177.93 | 12.03 | 346.77 | 34.01 | 0 |
| P2 | 3 | 5 | 6 | 750 | 2 | 177 | 15 | 0.13 | 17.17 | 350.81 | 14.43 | 2 |
| P3 | 4 | 4 | 5 | 1667 | 2 | 351 | 18 | 86.41 | 15.30 | 265.26 | 65.52 | 2 |
| P4 | 5 | 6 | 7 | 2500 | 0 | 600 | 15 | 6.82 | 10.06 | 279.75 | 0.20 | 3 |
| P5 | 5 | 6 | 4 | 2500 | 2 | 450 | 25 | 114.06 | 11.86 | 309.25 | 20.28 | 1 |
| P6 | 4 | 4 | 8 | 1650 | 2 | 160 | 20 | 6.10 | 19.24 | 282.18 | 12.36 | 1 |
| P7 | 3 | 4 | 5 | 600 | 2 | 310 | 15 | 78.27 | 15.65 | 360.50 | 47.42 | 0 |
| P8 | 3 | 4 | 7 | 800 | 2 | 100 | 15 | 0.17 | 14.62 | 0.36 | 8.26 | 2 |

**Table B.** Plant functional group (PFG) description and species list. Groups were built based on species’ abiotic requirements and functional traits [6] and group descriptions were based on expert knowledge and group average functional trait values. Table adapted from Boulangeat *et al.* [5].

| **Group** | **Description** | **Species list** |
| --- | --- | --- |
| C1 | Thermophilous chamaephytes with long dispersal distance | *Rumex acetosella, Cotoneaster integerrimus, Potentilla neumanniana, Rubus idaeus, Rubus saxatilis, Valeriana montana, Lonicera caerulea, Helianthemum grandiflorum, Helianthemum nummularium, Anthyllis montana, Hippocrepis comosa, Achillea millefolium, Stachys recta, Teucrium chamaedrys, Thymus pulegioides.* |
| C2 | Alpine and subalpine chamaephyte species | *Rumex scutatus, Salix hastata, Saxifraga aizoides, Saxifraga oppositifolia, Helictotrichon sedenense, Leucanthemopsis alpina, Cerastium alpinum, Cerastium cerastoides, Cerastium latifolium, Cerastium pedunculatum, Cerastium uniflorum, Sempervivum arachnoideum, Vaccinium uliginosum microphyllum, Antennaria dioica, Thymus polytrichus, Artemisia umbelliformis eriantha, Artemisia umbelliformis.* |
| C3 | Chamaephytes with short dispersal distance | *Androsace pubescens, Androsace vitaliana, Primula hirsuta, Primula latifolia, Dryas octopetala, Salix herbacea, Salix reticulata, Salix retusa, Saxifraga bryoides, Saxifraga exarata, Eritrichium nanum, Noccaea rotundifolia, Pritzelago alpina, Gypsophila repens, Sagina glabra, Sagina saginoides, Silene acaulis, Silene acaulis bryoides, Sedum album, Sedum alpestre, Sedum dasyphyllum, Empetrum nigrum hermaphroditum, Rhododendron ferrugineum, Globularia cordifolia.* |
| C4 | Tall shrubs | *Amelanchier ovalis, Crataegus monogyna, Rosa pendulina, Salix laggeri, Juniperus communis, Alnus alnobetula, Lonicera xylosteum, Cornus sanguinea, Corylus avellana, Ribes petraeum.* |
| C5 | Mountainous to subalpine heath found in dry climates | *Arctostaphylos uva-ursi crassifolius, Calluna vulgaris, Hippocrepis emerus.* |
| C6 | Mountainous to subalpine heath found in wet climates | *Vaccinium myrtillus, Vaccinium vitis-idaea.* |
| H1 | Alpine species (shade-intolerant, and with short dispersal distance) | *Oxyria digyna, Polygonum viviparum, Ranunculus glacialis, Ranunculus kuepferi, Ranunculus montanus, Geum montanum, Geum reptans, Potentilla aurea, Potentilla erecta, Potentilla grandiflora, Saxifraga stellaris robusta, Linaria alpina, Carex capillaris, Carex curvula, Carex foetida, Carex frigida, Carex nigra, Carex panicea, Carex rupestris, Eriophorum latifolium, Eriophorum polystachion, Eriophorum scheuchzeri, Kobresia myosuroides, Trichophorum cespitosum, Juncus alpinoarticulatus, Juncus trifidus, Luzula alpinopilosa, Agrostis alpina, Agrostis rupestris, Alopecurus alpinus, Avenula versicolor versicolor, Festuca halleri halleri, Festuca quadriflora, Phleum alpinum, Poa alpina, Poa cenisia, Poa laxa, Doronicum grandiflorum, Trisetum distichophyllum, Athamanta cretensis, Hieracium glaciale, Leontodon montanus, Leontodon pyrenaicus helveticus, Taraxacum alpinum, Campanula cochleariifolia, Astragalus alpinus, Lotus alpinus, Trifolium alpinum, Trifolium pallescens, Achillea nana, Gentiana punctata, Arnica montana, Epilobium anagallidifolium, Plantago alpina.* |
| H2 | Mountainous species that tolerate nitrophilous soils and have long dispersal distance | *Rumex acetosa, Rumex pseudalpinus, Fragaria vesca, Galium aparine, Galium verum, Carex caryophyllea, Carex sempervirens, Agrostis capillaris, Agrostis stolonifera, Festuca nigrescens, Sesleria caerulea, Astrantia major, Leucanthemum vulgare, Carum carvi, Meum athamanticum, Chenopodium bonus-henricus, Lathyrus pratensis, Lotus corniculatus, Onobrychis montana, Trifolium montanum, Trifolium pratense, Geranium sylvaticum, Plantago media.* |
| H3 | Mountainous to lowland species found in wet niches and with long dispersal distance | *Ranunculus acris, Trollius europaeus, Urtica dioica, Aegopodium podagraria, Anthoxanthum odoratum, Arrhenatherum elatius elatius, Dactylis glomerata, Deschampsia cespitosa, Festuca rubra, Crepis pyrenaica, Poa pratensis, Taraxacum officinale, Heracleum sphondylium, Pimpinella major, Trifolium repens, Vicia cracca, Plantago lanceolata.* |
| H4 | Undergrowth and shade-tolerant species that do not tolerate full light | *Aconitum lycoctonum vulparia, Aruncus dioicus, Dryopteris dilatata, Dryopteris filix-mas, Athyrium filix-femina, Prenanthes purpurea.* |
| H5 | Mountainous to subalpine species that have a short dispersal distance and tolerate dry soils | *Pulsatilla alpina, Ranunculus bulbosus, Anthericum liliago, Luzula sieberi, Achnatherum calamagrostis, Agrostis agrostiflora, Briza media, Bromus erectus, Deschampsia flexuosa, Festuca acuminata, Festuca flavescens, Festuca laevigata, Festuca marginata gallica, Koeleria vallesiana, Phleum alpinum rhaeticum, Stipa eriocaulis eriocaulis, Trisetum flavescens, Leontodon autumnalis, Leontodon hispidus, Tolpis staticifolia, Festuca melanopsis, Hugueninia tanacetifolia, Laserpitium halleri, Laserpitium siler, Silene flos-jovis, Hypericum maculatum, Salvia pratensis, Epilobium dodonaei fleischeri.* |
| H6 | Tall plants typical of ‘megaphorbiaies’ that can form undergrowth | *Ranunculus aduncus, Cacalia alliariae, Saxifraga rotundifolia, Valeriana officinalis, Carex flacca, Cicerbita alpina, Luzula nivea, Avenula pubescens, Brachypodium rupestre, Calamagrostis varia, Festuca altissima, Melica nutans, Milium effusum, Molinia caerulea arundinacea, Poa nemoralis, Hieracium murorum, Hieracium prenanthoides, Senecio ovatus ovatus, Chaerophyllum aureum, Chaerophyllum villarsii, Cardamine pentaphyllos, Laserpitium latifolium, Knautia dipsacifolia, Mercurialis perennis, Gentiana lutea, Epilobium angustifolium.* |
| H7 | Plants species found in rocky habitats and undergrowth at all elevations | *Cacalia alpina, Cryptogramma crispa, Asplenium ramosum, Asplenium septentrionale septentrionale, Asplenium trichomanes quadrivalens, Equisetum arvense, Cystopteris fragilis, Gymnocarpium robertianum, Woodsia alpina, Hieracium pilosella, Homogyne alpina, Petasites albus, Tussilago farfara.* |
| H8 | Subalpine to alpine species not usually grazed and that have a short dispersal distance | *Cacalia leucophylla, Cirsium spinosissimum, Omalotheca supina, Murbeckiella pinnatifida pinnatifida, Gentiana alpina.* |
| H9 | Short subalpine to alpine species with long dispersal distance | *Anthoxanthum odoratum nipponicum, Nardus stricta, Poa supina, Silene vulgaris prostrata.* |
| H10 | Mountainous species with long dispersal distance and shade tolerance | *Heracleum sphondylium elegans.* |
| P1 | Thermophilous pioneer trees (deciduous trees and pines) | *Prunus avium, Sorbus aria, Sorbus aucuparia, Sorbus mougeotii, Pinus cembra, Pinus sylvestris.* |
| P2 | Small deciduous pioneer trees (e.g. colonising riversides) | *Populus tremula, Salix daphnoides.* |
| P3 | Tall forest edge trees | *Tilia platyphyllos, Acer pseudoplatanus, Fraxinus excelsior.* |
| P4 | Tall pioneer (larch) | *Larix decidua.* |
| P5 | Late succession trees found in wet climates | *Picea abies, Fagus sylvatica.* |
| P6 | Intermediate succession trees found in dry climates | *Pinus uncinata, Betula pendula.* |
| P7 | Small forest edge trees | *Acer opalus, Acer campestre campestre.* |
| P8 | Small pioneer found in cold climates (white birch) | *Betula pubescens.* |

**Table C.** Plant functional group (PFG) relative abundances before disturbances. PFG abundances were aggregated by life form (colour-coded) and averaged by community type (unmanaged forests, managed and unmanaged grasslands) across the three simulation replicates.

|  | **PFG** | **Mean rel. abundance** | **std. dev** |
| --- | --- | --- | --- |
| Unmanaged | C1 | 7.83E-04 | 3.85E-05 |
| forests | C2 | 2.65E-03 | 1.45E-04 |
|  | C3 | 9.36E-04 | 3.58E-05 |
|  | C4 | 9.74E-02 | 4.53E-05 |
|  | C5 | 3.13E-02 | 5.98E-05 |
|  | C6 | 2.91E-02 | 4.29E-05 |
|  | H1 | 1.27E-03 | 6.37E-05 |
|  | H2 | 1.51E-03 | 3.61E-05 |
|  | H3 | 4.38E-06 | 1.24E-06 |
|  | H4 | 5.84E-02 | 8.35E-05 |
|  | H5 | 3.10E-03 | 1.41E-04 |
|  | H6 | 7.69E-02 | 1.13E-04 |
|  | H7 | 6.51E-02 | 6.16E-05 |
|  | H8 | 6.96E-04 | 5.88E-05 |
|  | H9 | 9.64E-04 | 2.80E-05 |
|  | H10 | 1.55E-02 | 6.56E-05 |
|  | P1 | 1.61E-01 | 1.64E-04 |
|  | P2 | 1.03E-01 | 2.47E-04 |
|  | P3 | 1.37E-01 | 2.04E-04 |
|  | P4 | 4.22E-03 | 1.36E-04 |
|  | P5 | 1.35E-01 | 7.77E-05 |
|  | P6 | 2.80E-03 | 8.40E-05 |
|  | P7 | 6.66E-02 | 8.00E-05 |
|  | P8 | 4.92E-03 | 1.52E-04 |
| Managed | C1 | 4.15E-03 | 3.22E-05 |
| grasslands/prairies | C2 | 4.73E-03 | 1.49E-05 |
|  | C3 | 1.92E-01 | 2.31E-04 |
|  | C4 | 3.99E-04 | 7.40E-07 |
|  | C5 | 2.91E-02 | 1.22E-04 |
|  | C6 | 2.59E-02 | 6.16E-05 |
|  | H1 | 2.87E-03 | 5.00E-06 |
|  | H2 | 1.29E-02 | 4.99E-05 |
|  | H3 | 6.02E-04 | 1.88E-04 |
|  | H4 | 6.06E-04 | 1.36E-05 |
|  | H5 | 5.99E-03 | 2.76E-05 |
|  | H6 | 5.74E-03 | 2.61E-05 |
|  | H7 | 5.25E-01 | 6.98E-04 |
|  | H8 | 1.66E-01 | 3.79E-04 |
|  | H9 | 1.08E-02 | 3.91E-05 |
|  | H10 | 6.37E-03 | 2.47E-05 |
|  | P1 | 6.26E-05 | 9.44E-07 |
|  | P2 | 2.26E-05 | 8.30E-07 |
|  | P3 | --- | --- |
|  | P4 | --- | --- |
|  | P5 | 2.47E-03 | 1.24E-05 |
|  | P6 | 4.58E-03 | 8.39E-06 |
|  | P7 | 1.45E-04 | 1.70E-06 |
|  | P8 | --- | --- |
| Unmanaged | C1 | 2.94E-04 | 1.91E-05 |
| grasslands/prairies | C2 | 2.25E-01 | 2.04E-04 |
|  | C3 | 1.85E-01 | 1.21E-04 |
|  | C4 | 4.48E-06 | 5.38E-07 |
|  | C5 | 5.90E-04 | 2.29E-05 |
|  | C6 | 5.85E-02 | 1.31E-04 |
|  | H1 | 1.42E-01 | 5.76E-05 |
|  | H2 | 1.52E-02 | 1.09E-04 |
|  | H3 | 1.60E-07 | 1.99E-07 |
|  | H4 | 2.72E-06 | 3.95E-07 |
|  | H5 | 9.43E-03 | 9.16E-05 |
|  | H6 | 2.79E-04 | 1.01E-05 |
|  | H7 | 9.09E-02 | 6.33E-05 |
|  | H8 | 1.72E-01 | 1.59E-04 |
|  | H9 | 7.43E-02 | 3.45E-05 |
|  | H10 | 9.92E-04 | 2.91E-05 |
|  | P1 | 2.93E-05 | 5.82E-06 |
|  | P2 | 8.59E-06 | 1.40E-06 |
|  | P3 | --- | --- |
|  | P4 | 1.63E-02 | 1.35E-04 |
|  | P5 | 9.80E-05 | 2.48E-07 |
|  | P6 | 1.97E-03 | 5.80E-05 |
|  | P7 | 1.28E-07 | 1.46E-07 |
|  | P8 | 6.98E-03 | 3.78E-05 |

**Table D**. Model accuracy metrics. Adapted from Boulangeat *et al.* [5] and Barros *et al.* [11].

|  |  | Sensitivity |  |  | Specificity |  |  | Error rate |  |
| --- | --- | --- | --- | --- | --- | --- | --- | --- | --- |
| PFG | FATE-HD  w/ drought | FATE-HD  base model | HSM | FATE-HD  w/ drought | FATE-HD  base model | HSM | FATE-HD  w/ drought | FATE-HD  base model | HSM |
| C1 | 0.76 | 0.82 | 0.87 | 0.45 | 0.42 | 0.51 | 0.51 | 0.53 | 0.44 |
| C2 | 0.84 | 0.84 | 0.00 | 0.57 | 0.57 | 1.00 | 0.38 | 0.38 | 0.19 |
| C3 | 0.94 | 0.94 | 0.96 | 0.49 | 0.47 | 0.47 | 0.31 | 0.32 | 0.31 |
| C4 | 0.42 | 0.4 | 0.75 | 0.88 | 0.9 | 0.64 | 0.21 | 0.19 | 0.34 |
| C5 | 0.26 | 0.26 | 0.52 | 0.75 | 0.75 | 0.57 | 0.31 | 0.31 | 0.44 |
| C6 | 0.60 | 0.59 | 0.64 | 0.57 | 0.57 | 0.60 | 0.43 | 0.43 | 0.40 |
| H1 | 0.84 | 0.90 | 0.00 | 0.41 | 0.36 | 1.00 | 0.44 | 0.45 | 0.36 |
| H2 | 0.91 | 0.92 | 0.93 | 0.12 | 0.12 | 0.20 | 0.67 | 0.67 | 0.60 |
| H3 | 0.10 | 0.06 | 0.78 | 0.91 | 0.95 | 0.44 | 0.21 | 0.18 | 0.51 |
| H4 | 0.17 | 0.18 | 0.57 | 0.87 | 0.89 | 0.62 | 0.21 | 0.19 | 0.38 |
| H5 | 0.88 | 0.88 | 0.88 | 0.25 | 0.26 | 0.32 | 0.59 | 0.58 | 0.54 |
| H6 | 0.64 | 0.65 | 0.61 | 0.55 | 0.55 | 0.59 | 0.43 | 0.43 | 0.40 |
| H7 | 0.63 | 0.66 | 0.72 | 0.34 | 0.36 | 0.33 | 0.60 | 0.57 | 0.59 |
| H8 | 0.52 | 0.58 | 0.52 | 0.67 | 0.60 | 0.71 | 0.34 | 0.40 | 0.30 |
| H9 | 0.24 | 0.25 | 0.63 | 0.70 | 0.66 | 0.58 | 0.33 | 0.36 | 0.42 |
| H10 | 0.47 | 0.46 | 0.52 | 0.59 | 0.59 | 0.61 | 0.42 | 0.42 | 0.40 |
| P1 | 0.35 | 0.32 | 0.75 | 0.88 | 0.89 | 0.56 | 0.15 | 0.15 | 0.43 |
| P2 | 0.31 | 0.3 | 0.56 | 0.84 | 0.85 | 0.64 | 0.17 | 0.17 | 0.36 |
| P3 | 0.10 | 0.1 | 0.64 | 0.97 | 0.97 | 0.66 | 0.06 | 0.06 | 0.34 |
| P4 | 0.35 | 0.35 | 0.62 | 0.77 | 0.77 | 0.66 | 0.28 | 0.27 | 0.34 |
| P5 | 0.57 | 0.57 | 0.60 | 0.84 | 0.84 | 0.78 | 0.17 | 0.17 | 0.22 |
| P6 | 0.42 | 0.46 | 0.63 | 0.74 | 0.68 | 0.47 | 0.27 | 0.32 | 0.53 |
| P7 | 0.08 | 0.08 | 0.22 | 0.93 | 0.94 | 0.81 | 0.09 | 0.08 | 0.21 |
| P8 | 0.15 | 0.15 | 0.06 | 0.90 | 0.9 | 0.98 | 0.12 | 0.12 | 0.04 |

**Table E.** Results of the analyses of variance (ANOVAs) including ‘null comparisons’ as control treatment. Type III ANOVAs were used to assess whether the effects of drought scenarios and their interaction with community/management types on hypervolume metrics significantly differed from a *no-changes* scenario (i.e. null comparisons, included as the control in ‘*scenarios*’). Response variables (overlap, mean distance and size changes) were transformed when necessary to obey linear model assumptions. In model formulas ‘*’ denotes the inclusion of main effects and their interaction in the model. For instance, *overlap ~ scenario*community* is to be understood as *overlap ~ scenario + community + scenario:community*, with ‘:’ denoting the interaction between two factors. Asterisks a significance of *F-test* statistics at *p-value* < 0.001. ‘df’ stands for degrees of freedom and ‘Sum sq.’ for sums of squares.

|  |  | df | Sum sq. | F value |  |
| --- | --- | --- | --- | --- | --- |
| log-overlap ~ scenario*community | scenario | 3 | 5438.40 | 142413.50 | * |
| (managed grasslands excluded) | community | 1 | 490.90 | 38564.10 | * |
|  | scenario:community | 3 | 219.60 | 5751.50 | * |
|  | residuals | 1992 | 25.40 |  |  |
|  |  |  |  |  |  |
| log-overlap ~ scenario*management | management | 1 | 5047.60 | 155986.00 | * |
| (forests excluded) | scenario | 3 | 8313.20 | 85635.00 | * |
|  | scenario:management | 3 | 564.90 | 5819.00 | * |
|  | residuals | 1992 | 64.50 |  |  |
|  |  |  |  |  |  |
| mean distance ~ scenario*community | scenario | 3 | 12.74 | 438769.90 | * |
| (managed grasslands excluded) | community | 1 | 0.30 | 30812.80 | * |
|  | scenario:community | 3 | 0.11 | 3881.60 | * |
|  | residuals | 1992 | 0.02 |  |  |
|  |  |  |  |  |  |
| mean distance ~ scenario*management | management | 1 | 10.55 | 1007299.00 | * |
| (forests excluded) | scenario | 3 | 8.25 | 262424.00 | * |
|  | scenario:management | 3 | 0.94 | 29968.00 | * |
|  | residuals | 1992 | 0.02 |  |  |
|  |  |  |  |  |  |
| size changes ~ scenario*community | community | 1 | 3.30 | 148972.60 | * |
| (managed grasslands excluded) | scenario | 3 | 1.89 | 28373.00 | * |
|  | scenario:community | 3 | 0.45 | 6829.80 | * |
|  | residuals | 199 | 0.04 |  |  |
|  |  |  |  |  |  |
| size changes ~ scenario*management | management | 1 | 134.49 | 3197810.79 | * |
| (forests excluded) | scenario:management | 3 | 0.79 | 6228.66 | * |
|  | scenario | 3 | 0.05 | 391.29 | * |
|  | residuals | 1992 | 0.08 | 142413.50 |  |

**Table F.** Results of the analyses of variance (ANOVAs) when excluding null comparisons. In this case, we used Type I ANOVAs to assess the significant differences between drought scenarios and community/management types (and their interactions) on hypervolume metrics. Refer to the legend in Table E for symbology and abbreviations.

|  |  | df | Sum sq. | F value |  |
| --- | --- | --- | --- | --- | --- |
| log-overlap ~ scenario*community | community | 1 | 935.94 | 76800.00 | * |
| (managed grasslands excluded) | scenario:community | 2 | 139.14 | 5708.50 | * |
|  | scenario | 2 | 129.41 | 5309.60 | * |
|  | residuals | 1794 | 21.86 |  |  |
|  |  |  |  |  |  |
| log-overlap ~ scenario*management | management | 1 | 5270.90 | 151214.40 | * |
| (forests excluded) | scenario | 2 | 2174.50 | 31191.40 | * |
|  | scenario:management | 2 | 552.80 | 7928.90 | * |
|  | residuals | 1794 | 62.50 |  |  |
|  |  |  |  |  |  |
| mean distance ~ scenario*community | community | 1 | 0.59 | 60428.70 | * |
| (managed grasslands excluded) | scenario:community | 2 | 0.06 | 2891.90 | * |
|  | scenario | 2 | 0.03 | 1779.20 | * |
|  | residuals | 1794 | 0.02 |  |  |
|  |  |  |  |  |  |
| mean distance ~ scenario*management | management | 1 | 7.18 | 681808.98 | * |
| (forests excluded) | scenario | 2 | 0.22 | 10429.44 | * |
|  | scenario:management | 2 | 0.00 | 150.69 | * |
|  | residuals | 1794 | 0.02 |  |  |
|  |  |  |  |  |  |
| size changes ~ scenario*community | community | 1 | 6.02 | 254459.40 | * |
| (managed grasslands excluded) | scenario | 2 | 0.04 | 912.70 | * |
|  | scenario:community | 2 | 0.01 | 287.60 | * |
|  | residuals | 1794 | 0.04 |  |  |
|  |  |  |  |  |  |
| size changes ~ scenario*management | management | 1 | 168.34 | 4040070.00 | * |
| (forests excluded) | scenario:management | 2 | 0.06 | 707.25 | * |
|  | scenario | 2 | 0.02 | 291.22 | * |
|  | residuals | 1794 | 0.08 |  |  |
