# Supplementary material for: Drought effects on the stability of forest-grassland ecotones under gradual climate change
Source: PLoS One. 2018 Oct 24;13(10):e0206138. doi: 10.1371/journal.pone.0206138 (PMC6200273; doi:10.1371/journal.pone.0206138)
Supplement: S4 Appendix — (DOCX) [file pone.0206138.s004.docx]

**S4 Appendix - Supplementary figures**


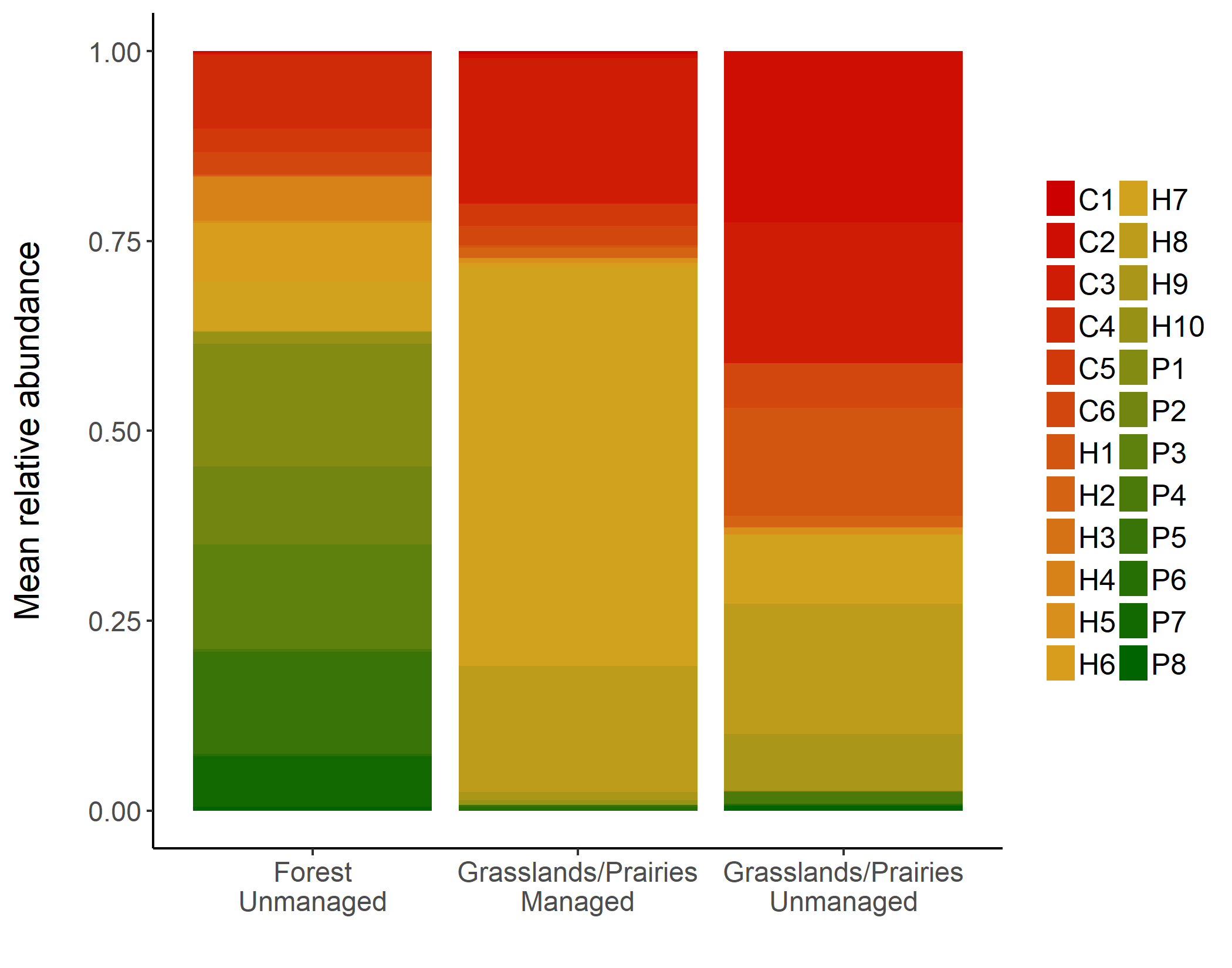


**Figure A.** Plant functional group (PFG) relative abundances before disturbances. Bars show PFG relative abundances at the end of the initialisation phase averaged by community type (unmanaged forests, managed and unmanaged grasslands) across the three simulation replicates. See Table C in S3 Appendix for average values and their respective standard deviations.


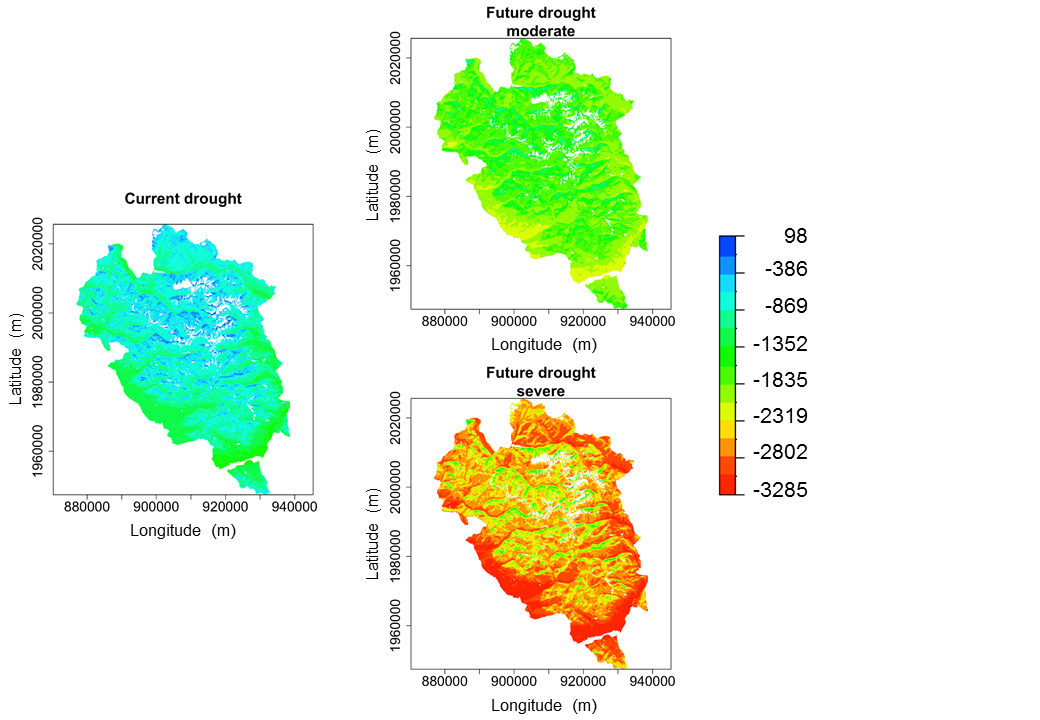


**Figure B.** Current and future drought intensity (*Din*) maps. Figure adapted from Barros *et al.* [11].

**
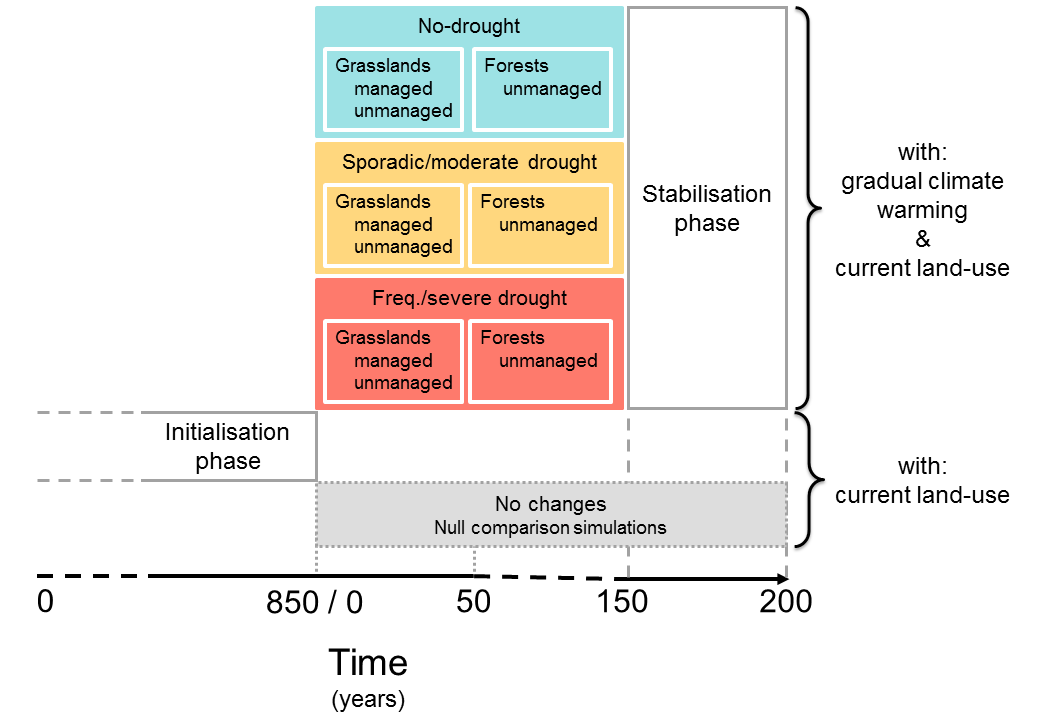
****Figure C.** Scheme of simulation experiment. Scheme of the simulation experiment used to understand the effects of climate change and drought regimes on departures from stability of grassland and forest communities. Note the effects of different management regimes were only assessed for grassland communities, which lead to an unbalanced design. Simulations – initialisation phase + scenario phases (blue, yellow and red boxes) + stabilisation phase – were repeated 3 times. Null comparison simulations were repeated 100 times, each starting from the first repetition of the initialisation phase.

**
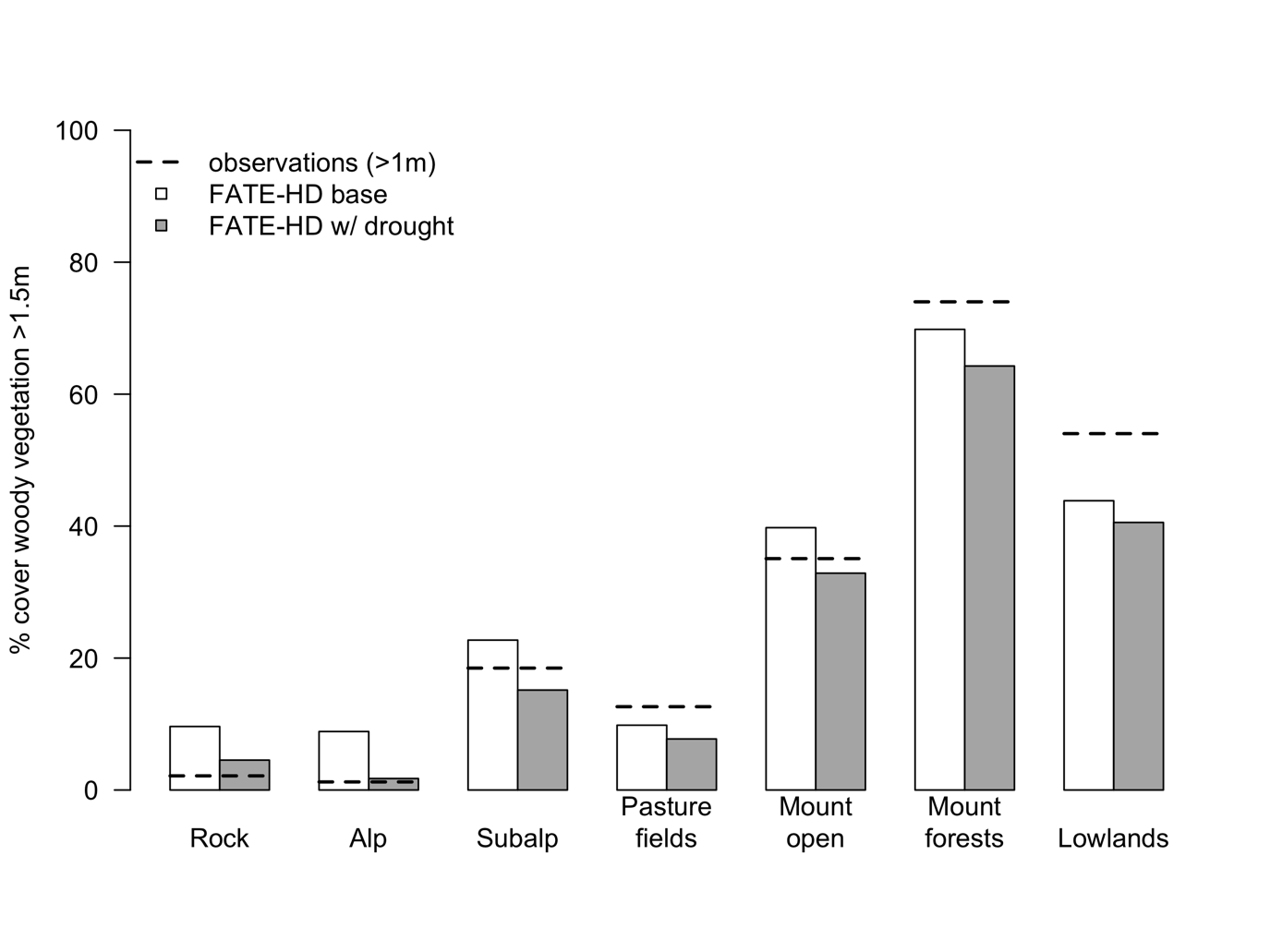
**

**Figure D.** Comparison of simulated tree cover (strata >1.5m) at the end of the validation simulation with predictions from the FATE-HD base model version, and with observed tree cover (woody strata >1m). Percentages shown refer to the number of pixels of each habitat where tree cover was present. Taken from Barros *et al.* [11].

**
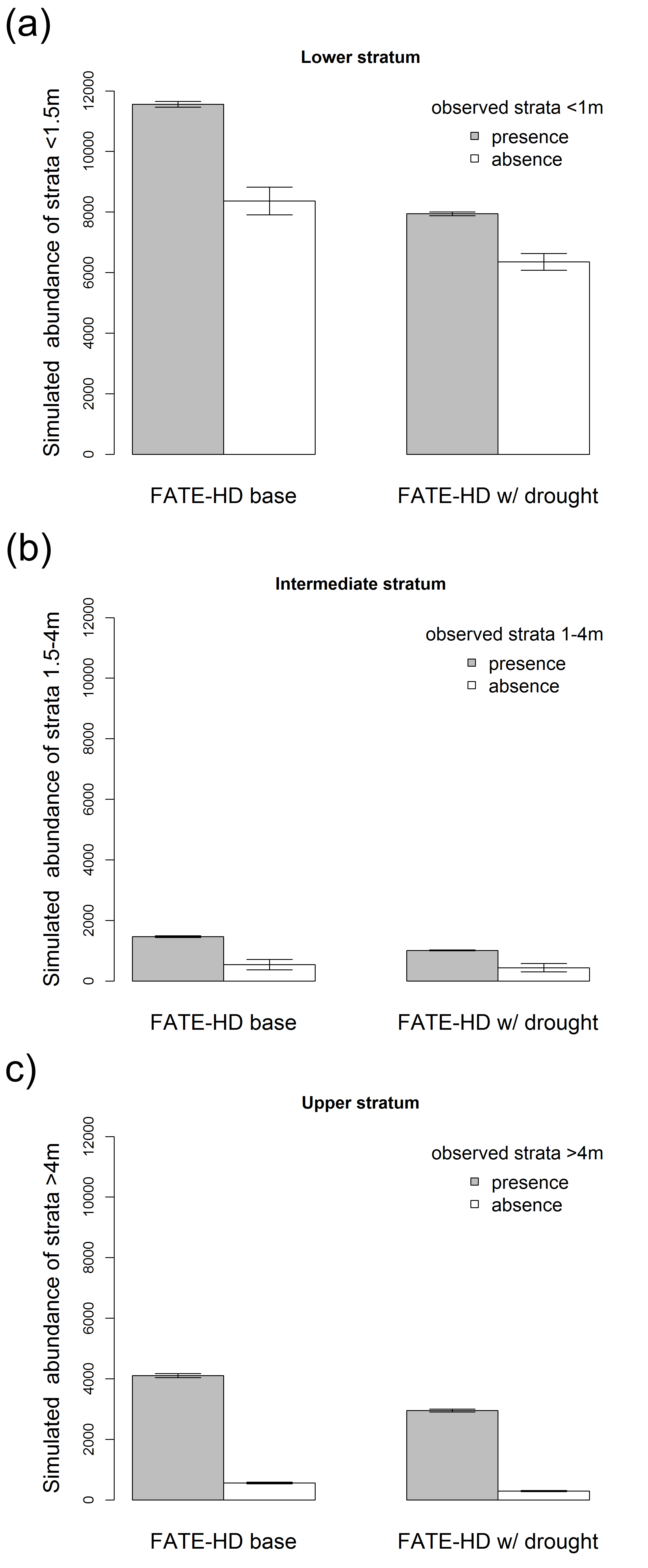
**

**Figure E.** Comparison of simulated abundances of three vegetation height strata in pixels where the strata were observed to be present (grey) and absent (white). Simulated abundances are shown for the FATE-HD base model and the version with drought. Taken from Barros et al. [11].


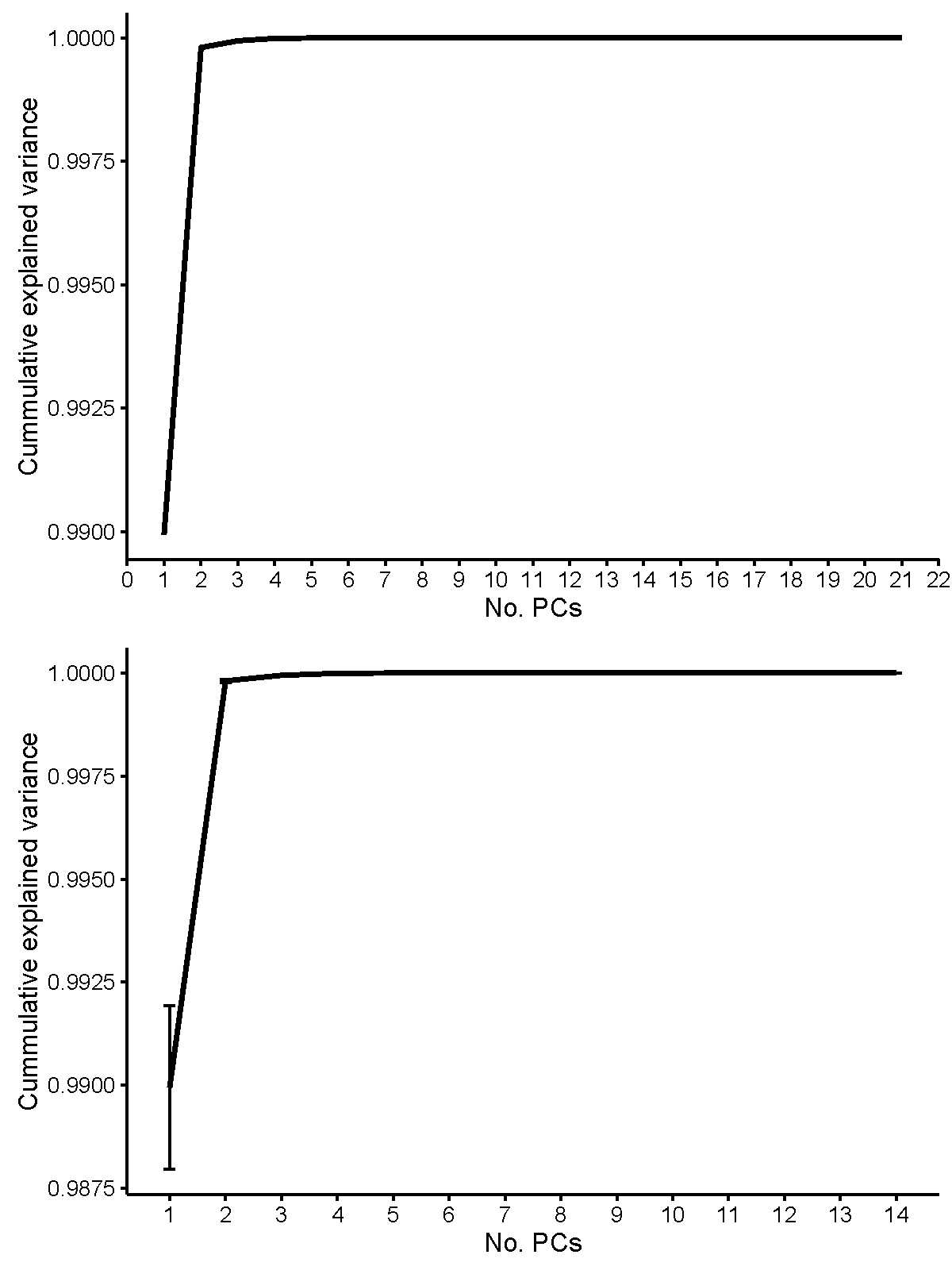


**Figure F.** Average proportion of explained variance accumulated across principal components (PCs). We show here the cumulative explained variance for an increasing number of PCs, averaged across all the calculated Principal Components Analyses (one per pair of current and future hypervolumes), except for those from null comparisons. Error bars represent standard errors.

**
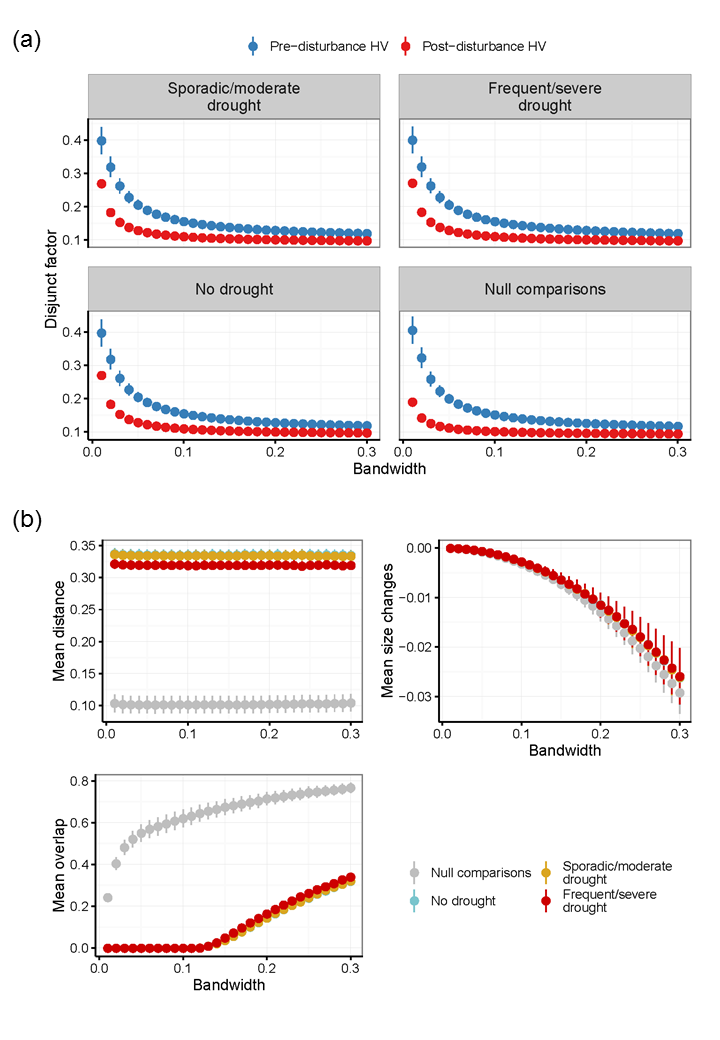
**

**Figure G.** Bandwidth sensitivity analysis. Effect of bandwidth values on *(a)* hypervolumes’ disjunct factor values and *(b)* hypervolume comparison metrics. Results of the bandwidth sensitivity analyses shown here are averaged across plant community and management combinations, and across repetitions. Vertical bars indicate standard errors. Only the first 3 repetitions of null comparison simulations were used.


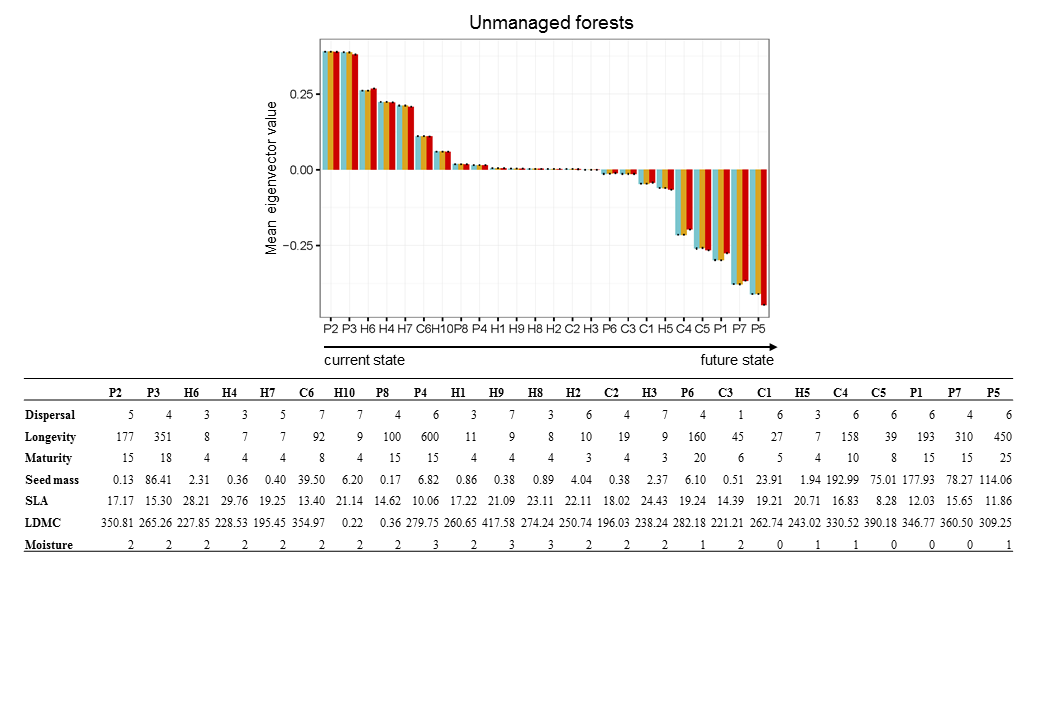


**Figure H.** PFGs eigenvalues on the first principal component of forest PCAs. One PCA was calculated per drought scenario (‘no drought’ in blue, ‘sporadic and moderate drought’ in yellow and ‘frequent and severe drought’ in red), including the last 50 years of the initialisation phase from which the ‘current’ state was defined. Corresponding PFG trait values are shown in the same order as the figure. Only the traits with correlations ≥ 0.8 with the first principal component, across all scenarios, are shown. For remaining trait values see Table A in S3 Appendix.


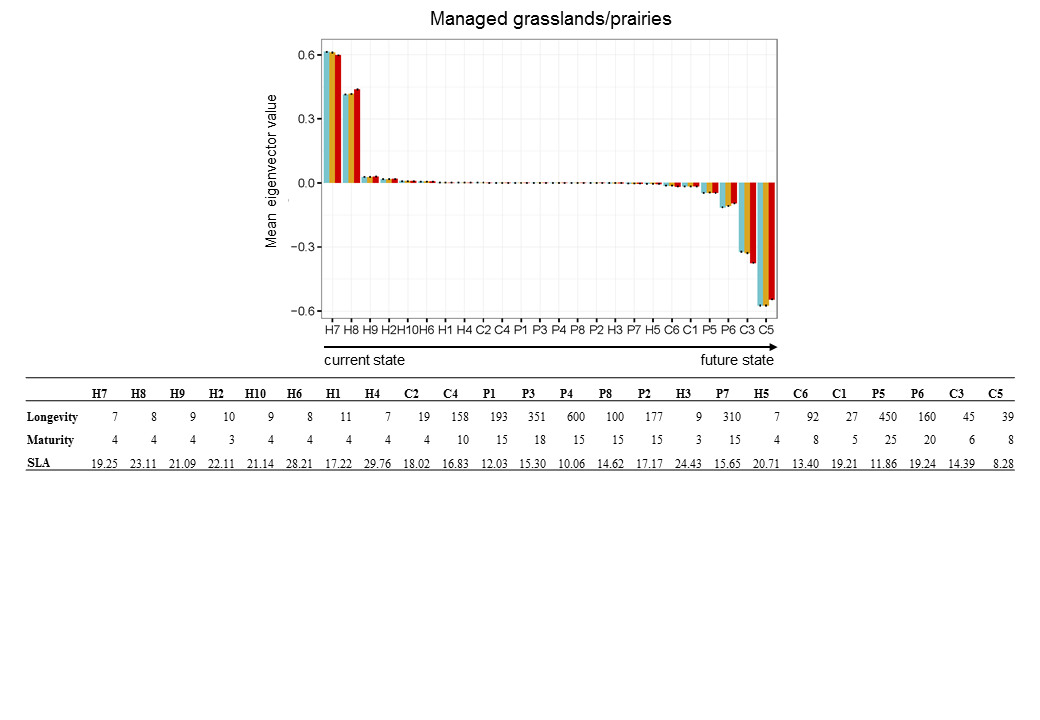


**Figure I.** PFGs eigenvalues on the first principal component of managed grasslands PCAs. See legend in Fig H for symbology and further details.


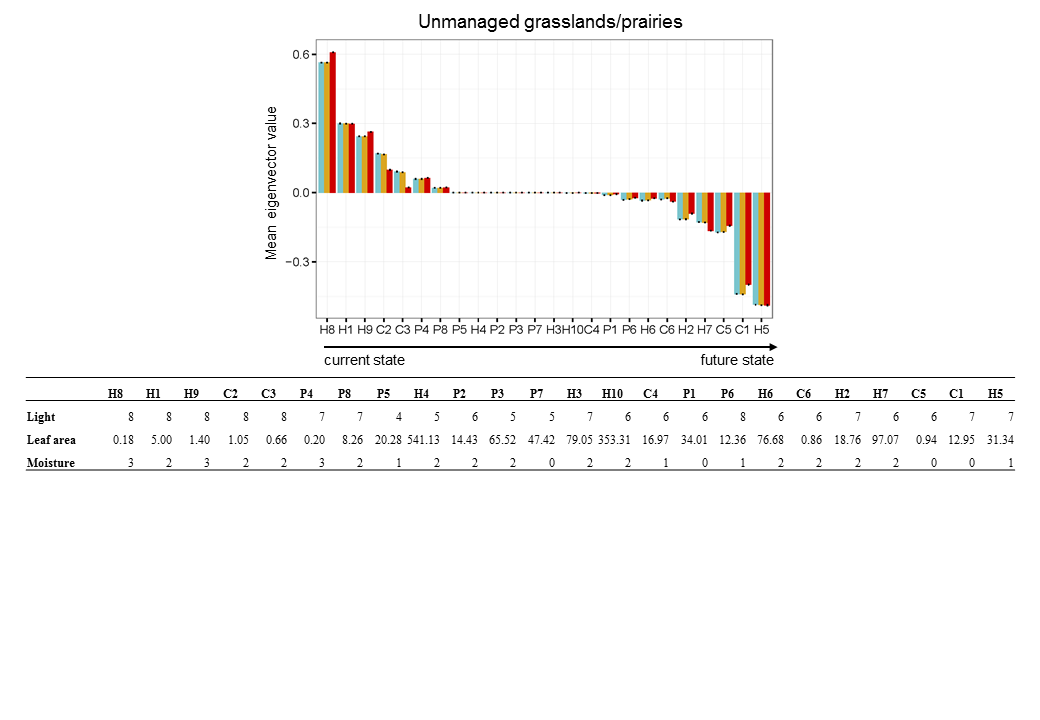


**Figure J.** PFGs eigenvalues on the first principal component of unmanaged grasslands PCAs. See legend in Fig H for symbology and further details.
